# Cost-effectiveness of palbociclib in early breast cancer patients with a high risk of relapse: Results from the PENELOPE-B trial

**DOI:** 10.3389/fonc.2022.886831

**Published:** 2022-09-05

**Authors:** Katya Galactionova, Sibylle Loibl, Paola Salari, Frederik Marmé, Miguel Martin, Michael Untch, Hervé R. Bonnefoi, Sung-Bae Kim, Harry D. Bear, Nicole McCarthy, Karen A. Gelmon, José A. García-Sáenz, Catherine M. Kelly, Toralf Reimer, Masakazu Toi, Hope S. Rugo, Michael Gnant, Andreas Makris, Nicole Burchardi, Matthias Schwenkglenks

**Affiliations:** ^1^ Institute of Pharmaceutical Medicine (ECPM), University of Basel, Basel, Switzerland; ^2^ German Breast Group, Neu-Isenburg, Germany; ^3^ Medical Faculty Mannheim, Heidelberg University, University Hospital Mannheim, Mannheim, Germany; ^4^ Instituto de Investigacion Sanitaria Gregorio Marañon, Centro de Investigación Biomédica en Red Cáncer (CIBERONC), Universidad Complutense, Madrid, Spain; ^5^ Spanish Breast Cancer Group, Grupo Español de Investigación en Cáncer de Mama (GEICAM), Madrid, Spain; ^6^ Department of Obstetrics and Gynaecology, Helios Kliniken Berlin-Buch, Berlin, Germany; ^7^ Department of Medical Oncology, Institut Bergonié and Université de Bordeaux Institut National de la Santé et de la Recherche Médicale (INSERM) U916, Bordeaux, France; ^8^ Asan Medical Center, University of Ulsan College of Medicine, Seoul, South Korea; ^9^ Division of Surgical Oncology, Massey Cancer Center, Virginia Commonwealth University, Virginia Commonwealth University (VCU) Health, Richmond, VA, United States; ^10^ Australia and New Zealand Breast Cancer Trials Group, Newcastle, NSW, Australia; ^11^ Department of Medical Oncology, University of Queensland, Brisbane, QLD, Australia; ^12^ BC Cancer, Vancouver, BC, Canada; ^13^ Instituto de Investigación Sanitaria del Hospital Clinico San Carlos (IdISSC), Madrid, Spain; ^14^ Grupo Español de Investigación en Cáncer de Mama (GEICAM), Madrid, Spain; ^15^ Mater Private Hospital, Cancer Trials Ireland, Dublin, Ireland; ^16^ Department of Obstetrics and Gynecology, University of Rostock, Rostock, Germany; ^17^ Breast Surgery, Graduate School of Medicine, Kyoto University, Kyoto, Japan; ^18^ University of California San Francisco Comprehensive Cancer Center, San Francisco, CA, United States; ^19^ Comprehensive Cancer Center, Medical University of Vienna, Vienna, Austria; ^20^ Institute of Cancer Research, London, United Kingdom

**Keywords:** Penelope-B, Palbociclib, CDKi, (postneo)adjuvant, early breast cancer, cost-effectiveness, Germany

## Abstract

**Background:**

Patients with hormone receptor-positive, HER2-negative breast cancer who have residual invasive disease after neoadjuvant chemotherapy (NACT) are at a high risk of relapse. PENELOPE-B was a double-blind, placebo-controlled, phase III trial that investigated adding palbociclib (PAL) for thirteen 28-day cycles to adjuvant endocrine therapy (ET) in these patients. Clinical results showed no significant improvement in invasive disease-free survival with PAL.

**Methods:**

We performed a pre-planned cost-effectiveness analysis of PAL within PENELOPE-B from the perspective of the German statutory health insurance. Health-related quality of life scores, collected in the trial using the EQ-5D-3L instrument, were converted to utilities based on the German valuation algorithm. Resource use was valued using German price weights. Outcomes were discounted at 3% and modeled with mixed-level linear models to adjust for attrition, repeated measurements, and residual baseline imbalances. Subgroup analyses were performed for key prognostic risk factors. Scenario analyses addressed data limitations and evaluated the robustness of the estimated cost-effectiveness of PAL to methodological choices.

**Results:**

The effects of PAL on quality-adjusted life years (QALYs) were marginal during the active treatment phase, increasing thereafter to 0.088 (95% confidence interval: −0.001; 0.177) QALYs gained over the 4 years of follow-up. The incremental costs were dominated by PAL averaging EUR 33,000 per patient; costs were higher in the PAL arm but not significantly different after the second year. At an incremental cost-effectiveness ratio of EUR 380,000 per QALY gained, PAL was not cost-effective compared to the standard-of-care ET. Analyses restricted to Germany and other subgroups were consistent with the main results. Findings were robust in the scenarios evaluated.

**Conclusions:**

One year of PAL added to ET is not cost-effective in women with residual invasive disease after NACT in Germany.

## Introduction

Breast cancer is the most frequent cancer among women in Europe. In 2020, about 355,000 new cases were diagnosed, with nearly 95,000 women dying of breast cancer in EU-27 that year (1). About 90% of new breast cancer patients in EU-27 countries are diagnosed at an early stage (2), of which, approximately a third will develop advanced or metastatic disease later in life (3). Prognosis depends on the number of positive axillary nodes, tumor size, tumor grade, lymphatic and vascular invasion, expression of estrogen (ER+) and progesterone receptors, and human epidermal growth factor 2 (HER2) status (4, 5).

In recent years, novel cancer treatments led to patient-relevant improvements in treatment outcomes. In particular, cyclin-dependent kinase (CDK) 4/6 inhibitors, including palbociclib (PAL), combined with endocrine therapy (ET) showed impressive efficacy in ER+ advanced breast cancer (6–8). In hormone receptor-positive breast cancer patients, CDKs modulate cell cycle entry and progression in response to growth signals (9, 10). Inhibition of these kinases with PAL could enhance the activity of other anticancer drugs.

PENELOPE-B follows a series of studies that established the efficacy of PAL in metastatic breast cancer (11). In 2015, the US FDA and, later, also the EMA approved PAL for use in combination with ET for first-line and, subsequently, second-line treatment of postmenopausal women with locally advanced or metastatic disease (12, 13). Since then, several recent and ongoing trials, including PENELOPE-B, have sought to demonstrate its efficacy also in high-risk ER+, HER2-, early breast cancer patients with residual disease after neoadjuvant chemotherapy (NACT) (14–17).

In PENELOPE-B, PAL added to standard adjuvant ET did not statistically improve invasive disease-free survival (iDFS) or overall survival (OS) compared to placebo (16). Findings on these patient-level outcomes expanded the clinical evidence base but were not sufficient to conclude on the value of PAL against competing claims for healthcare resources (18). These value judgments, addressed within the cost-effectiveness framework, integrate societal health state values through the use of preference-based health-related quality of life (HRQoL) measures and costs, thus reflecting efficiency and equity (18, 19). An intervention that does not lead to a meaningful benefit in terms of survival may nonetheless be a good value for money if it leads to a better HRQoL or changes in care-seeking that reduce overall spending in the patient group targeted. Toward this end, we present further, pre-planned analyses of the trial data on the effects of PAL on HRQoL, medical resource use, and cost of care, and address its cost-effectiveness compared to ET alone.

## Materials and methods

We performed a within-trial cost-effectiveness analysis of PAL+ET in PENELOPE-B from the perspective of the German statutory health insurance. Information on survival, disease progression, medical resource use, and HRQoL based on the European Quality of Life-5 Dimensions-3 Level (EQ-5D-3L) instrument was collected within the trial. Price weights were obtained from published national databases and the literature (20–25). A validated German valuation algorithm for ED-5D-3L was used to derive quality-adjusted life years (QALYs) (26). Mixed-level models (27) were used to adjust for missing values, stratification, and potential residual imbalances between the study arms at baseline. The evaluation was restricted to a within-trial horizon with a maximum follow-up (FU) of up to 6 years. Incremental costs and effects, discounted at 3%, were compared in each year and cumulatively over the duration of FU. Scenario analyses addressed data limitations and evaluated the robustness of the estimated cost-effectiveness of PAL to methodological choices. The main analysis was conducted on the intent-to-treat (ITT) population; subgroup analyses including by risk strata and country were also performed.

### Trial

PENELOPE-B (NCT01864746) was a randomized, double-blind, placebo-controlled, phase III trial that investigated the effects of PAL in early HR+ and HER2- breast cancer patients aged 18 and above (28). Women were eligible if they had residual disease after at least 16 weeks of NACT, were at a high risk of relapse [clinical pathological staging-estrogen receptor grading (CPS-EG) score ≥ 3 or 2 and ypN+ (29)], and subsequently underwent a definitive surgery and/or radiation.

Patients were recruited between February 2014 and December 2017 from 221 centers in Germany, Spain, USA, France, Australia, South Korea, Ireland, Japan, Austria, and UK. Randomization was in 1:1 permuted blocks of alternating size stratified by risk, nodal involvement after surgery, Ki-67 status, age, and region to receive either PAL (125 mg, orally, once daily for 21 days, followed by 1 week off treatment for a total duration of thirteen 4-week cycles) or placebo in addition to adjuvant ET and other standard-of-care treatment according to local guidelines (28). Patients were followed up for a maximum of 6 years. The primary clinical end point of the trial was iDFS.

The trial was approved by the health authorities and ethics committees and conformed to ICH-GCP guidelines and the Declaration of Helsinki. Further details on the trial are available from Loibl et al. (16).

### End point

The primary end point for the health-economic sub-study was the incremental cost-effectiveness of PAL+ET expressed as a cost per QALY gained compared to ET (implies placebo+ET here and throughout). The secondary objective was to compare between the arms HRQoL, accrued QALYs, medical resource use, and direct medical costs. The outcomes were assessed yearly and cumulatively within the trial FU. No extrapolation was done due to the lack of clinical differences between the trial arms at the end of FU.

### QALYs

The EQ-5D-3L (30, 31) was used to score HRQoL. The questionnaire, asking patients to rank their mobility, self-care, usual activities, pain/discomfort, and anxiety/depression, was completed at baseline (30 days prior to randomization), and during FU visits: bi-monthly during PAL treatment, at end of treatment (EOT), every 6 months in years 2–4, and every 12 months thereafter. The EQ-5D-3L scores were converted to utilities using the German valuation algorithm (26). QALYs were estimated by combining the estimated utilities with time using the “area under the curve” approach (32). For patients who died in the trial, QALYs were set to 0 from the date of death until the end of planned FU.

### Medical resource use, price weights, and costs

Medical resource use recorded in the trial covered all care episodes including those related to conditions other than breast cancer. Care episodes occurring at the enrolling and treating medical centers were transferred from the patients’ medical records. Patient diaries were used as the basis for recording the intake of the study drug and care episodes (outpatient physician visits and hospitalizations) occurring outside of the enrolling and treating centers. Information on the medical resource use generally allowed characterization of care episodes with respect to the type of care received, the number of events since last FU, and their duration facilitating costing. Where information was recorded as free text (diagnostic screenings, physician visits, and hospitalizations), coding routines were developed to map these entries into line items that could be consistently costed. See **Appendix A1** for further details on recording of resource use in the trial and adjustments for costing.

2020 German price weights (i.e., unit costs) were used to value resources. Drug prices were based on the median listed retail price per tablet (20). Costs of radiotherapy per session were obtained from the literature (24). Physician visits were costed by specialty based on the average fee reported by the National Association of Statutory Health Insurance Physicians [Kassenärztliche Bundesvereinigung (KBV)] (21); 2018 unit costs (the most recent available at the time of analysis) were inflated to 2020 prices using the German gross domestic product deflator (33). Screenings and other diagnostic examinations (i.e., CT, MRI, mammogram), minor surgeries (i.e., biopsy), and lymph drainage massage were also costed from KBV data (22). Physiotherapy costs were based on costs of inpatient hospital rehabilitation from the German Pension Insurance (Deutsche Rentenversicherung) (23). Inpatient hospital stays were costed based on an average cost per day for different types of hospitalization derived by dividing the average cost per stay by the average length of stay from appropriate diagnosis-related groups (DRGs) in the German DRG system (25). See **Appendix A1** for unit costs and further details on derivation.

Only resources used after randomization (including postoperative treatments, care related to comorbidities, adverse events, and treatments for recurrent or secondary malignancies) were considered. Costs were calculated per care episode by multiplying the quantity of the resource line item with the respective price weight and then summed. For patients who died during FU, costs were set to 0 from the date of death until the end of planned FU.

### Missing values

Missing values were encountered due to partial response (item-level missingness), attrition (loss to FU or withdrawal from the study), and, to the extent that we produced estimates for a given length of FU (as opposed to average FU), missingness due to administrative censoring (i.e., patients followed up for less than 72 months given their date of enrolment). Each of these sources of missing information required its own strategy to address.

Instances of item-level missingness were relatively few, resulting in <1% of missing values in utility and resource use data. These were resolved with information borrowed from the available data or filled with assumptions informed by clinical experts (see **Table A4** in **Appendix A1** for details). Care episodes that took place in the periods of missed FU were deemed not to pose a significant problem for costs since these were covered during the next FU visit at which the patient was present (i.e., medical records reviewed and relevant information updated since the last visit). Missing utility values were linearly interpolated from the periods just before the missing value and just after. Missing baseline utility values were imputed from the FU visit at the start of the first treatment cycle since the two were on average <30 days apart (patients have only received the first dose of study drug in-between).

Patients lost to FU for multiple consecutive periods were censored at the date of the last FU present; data in subsequent periods in which FU was resumed were not used (<2% of patient-FU records). For patients who died, costs were censored at the time of the previous FU when the patient was alive to reflect missing information on expenditures prior to death. For consistency, data used in regression analyses were further censored to exclude partial year entries, i.e., censoring at the last complete yearly interval.

In total, attrition resulted in 20% of QALYs and 11% of costs missing (see **Table A6** in **Appendix A1**). Attrition was balanced between the study arms and increased from about 13% for QALYs and 7% for costs in the first year to as much as 23% and 38%, respectively, in year 3 before dropping again in the later years. Administrative censoring accounted for another 23% of missing values. Missingness varied by country and was strongly and positively associated with the time of enrollment and CPS-EG score ≥3 (**Tables A7–A9** in **Appendix A1**).

### Between-country heterogeneity

We tested and found no evidence of heterogeneity in outcomes among countries (see **Table A10** in **Appendix A1**) (34–36). Thus, the pooled result applies to all countries that participated in the trial, including Germany.

### Cost-effectiveness analysis

The main analysis was conducted on the ITT population at 4 years after randomization; results for years 5 and 6 could not be reliably estimated due to high administrative censoring.

As the starting point for the main analysis, mean differences in outcomes were calculated by arm and year of FU. These descriptive results were then compared to regression-adjusted mean differences estimated with mixed models for repeated measures (MMRM) (27). The models adjusted for stratification (37) and addressed missingness under the missing at random assumption (MAR) (27, 38) and potential residual imbalances at baseline (32). The effect of PAL was captured with an interaction between the arm assignment and the year of FU. We modeled residuals using an unstructured covariance matrix that implies independence between patients. All models controlled for risk stratification factors, baseline health utility, and country; cost models additionally controlled for breast cancer treatments received before randomization (first ET with tamoxifen, ovarian ablation with goserelin injections, mastectomy, and reconstruction surgery) and the number of health conditions with ongoing treatment (0, 1, 2, 3, and more). Average marginal effects by year were summed to produce cumulative incremental outcomes for different lengths of FU. Model specification tests are reported in **Tables A11** and **A12** in **Appendix A1**. Alternative specifications, allowing for correlation between outcomes using seemingly unrelated regressions (SUR) and adjusting for skewness using generalized linear models (GLMs), were tested in scenario analyses.

The incremental cost-effectiveness ratio (ICER) was calculated as the ratio of cumulative incremental costs to QALYs.

### Uncertainty and scenario analyses

The 95% confidence intervals for the incremental QALYs and costs were estimated with nonparametric bootstrap stratified by arm with 5,000 replications. Confidence intervals and *p*-values were calculated by pooling bootstrapped standard errors over the respective yearly intervals. Regression-adjusted bootstrapped incremental outcomes were plotted on the cost-effectiveness plane.

Scenario analyses addressed data limitations and evaluated the robustness of the estimated cost-effectiveness of PAL to methodological choices and in different populations of interest. We tested our strategy for dealing with missing values by relaxing some of the censoring rules and by using multiple imputation by chained equations (MICE) to impute missing values for each FU year (see **Appendix A2** for details on the implementation of MICE) (39, 40). Analyses using imputed data allowed us to further explore between- and within-patient correlations (38).

### Technical implementation

All analyses were implemented in Stata/SE version16.1 (41).

## Results

### Patient characteristics

The PENELOPE-B study population was previously described in Loibl et al. (16). For context, patient characteristics are reported in **Table A13** in **Appendix A3**. We briefly note that Germany recruited over a third of all patients. These differed somewhat from the full study population in the distribution of risk (relatively higher share with CPS-EG score ≥3 in PAL+ET arm, lower with Ki-67 ≤15%), breast cancer treatments at baseline (fewer mastectomies, fewer started ET before PAL or ET with tamoxifen, fewer on goserelin, and fewer hysterectomies), and other illnesses (one illness less chronic or ongoing).

### Descriptive results

Clinical results have been previously reported in Loibl et al. (16). To facilitate the interpretation of incremental effects of PAL on HRQoL and costs, we present unadjusted clinical events along with healthcare utilization summaries (**Table 1**). On average, about 23% of patients relapsed, less than 2% developed a secondary malignancy, and about 10% died during the FU period. These fractions were relatively higher in the German subpopulation, reflecting longer FU and differences in baseline characteristics. In both samples, the fraction reporting an event was higher in the ET arm compared to PAL+ET, although this difference was not statistically significant.

**Table 1:** Clinical events and utilization.

Population	All countries			Germany		
Arm	PAL + ET (*N* = 631)	ET (*N* = 619)	*p*-value	PAL + ET (*N* = 218)	ET (*N* = 214)	*p*-value
Clinical events						
FU, years^a^	4.13 ± 0.84	4.13 ± 0.84	0.958	4.35 ± 0.98	4.38 ± 0.95	0.802
Relapsed, %	22.7	23.3	0.801	29.2	31.6	0.586
Number of relapses, *n*	1.63 ± 1.06	1.68 ± 1.22	0.971	1.54 ± 1.06	1.73 ± 1.06	0.148
Developed a secondary malignancy, %	1.6	1.8	0.791	1.8	1.9	0.979
Died, %	9.8	11.1	0.446	16.4	17.2	0.830
Any service use by type, %						
PAL	99.7	0.8	<0.001	99.5	0.5	<0.001
Hospitalization	45.4	44.7	0.818	51.4	50.7	0.888
Screening	98.9	99.2	0.583	99.5	98.6	0.308
Physical examinations and specialist visits^b^	99.8	99.8	0.990	100.0	99.5	0.313
Targeted therapy	8.6	11.0	0.151	10.1	14.4	0.170
Hormone therapy	100.0	99.8	0.313	100.0	100.0	<0.001
Ovarian suppression^c^	23.3	25.5	0.367	15.1	20.0	0.184
Radiation therapy	5.7	5.8	0.939	7.3	8.4	0.690
Chemotherapy	13.2	10.7	0.171	15.1	14.9	0.941
Mental health or physiotherapy	4.0	4.2	0.836	9.2	7.9	0.637
Of those with any service use number of visits/days of therapy/number of pills, *n*						
PAL	324 ± 95	336 ± 63	0.947	325 ± 93	364 ± .^d^	0.604
Hospitalizations	7 ± 13	7 ± 11	0.828	9 ± 10	11 ± 12	0.135
Screening	7 ± 5	7 ± 4	0.164	8 ± 5	7 ± 5	0.104
Physical examinations and specialist visits^b^	33 ± 12	29 ± 11	<0.001	34 ± 13	29 ± 13	<0.001
Targeted therapy	172 ± 133	196 ± 154	0.369	186 ± 138	210 ± 183	0.655
Hormone therapy	766 ± 397	769 ± 421	0.821	747 ± 398	794 ± 463	0.143
Ovarian suppression^c^	19 ± 17	18 ± 17	0.641	13 ± 11	11 ± 12	0.281
Radiation therapy	17 ± 12	19 ± 13	0.531	18 ± 10	22 ± 15	0.557
Chemotherapy	228 ± 196	280 ± 212	0.054	201 ± 147	309 ± 222	0.046
Mental health or physiotherapy	32 ± 41	17 ± 13	0.349	37 ± 44	23 ± 10	0.924

Continuous variables are summarized, with a mean ± SD. Significance of differences in the number of clinical events and care episodes between the arms was assessed with Fisher’s exact test for binary, continuity-corrected chi-square test for categorical, and Wilcoxon test for continuous parameters. ^a^FU refers to the number of years between patient entry date and study end date irrespective of event; ^b^Excluding visits related to administration of ovarian suppression, including examinations by physicians, referral, and follow-up visits related to screenings and hospitalizations; ^c^Included goserelin or other luteinizing hormone-releasing hormone (LHRH) injections but not surgery or radiotherapy that were covered under the respective event types; ^d^SD missing since only one patient received PAL in ET arm in Germany. ET, endocrine therapy; FU, follow-up; PAL, palbociclib.

Most patients had at least one screening, one visit with a physician, and had taken at least one hormone therapy pill. About half had a hospitalization, and about a quarter received additional ovarian suppression injections. Other types of care were less common. Consistent with differences in clinical events, a higher fraction of patients in the German subpopulation were hospitalized, received targeted therapy, mental health services, and physiotherapy compared to the full study population. With the exception of PAL, there were no statistical differences between the arms in healthcare utilization. Furthermore, mean quantities by type of care were balanced except for PAL and physician visits (about five more in PAL+ET, incurred in the first year; see **Table A14** and **Figures A7–A12** in **Appendix A3** for additional tabulations).

In the full study population, over the average FU of about 2.8 years, patients in the PAL+ET arm gained an additional 0.07 discounted QALYs compared to the ET arm, and this difference was not significant (**Table 2**). In the German subpopulation, the relative gains were larger (0.23 discounted QALYs) and marginally significant. Differences in total costs were statistically significant and roughly equal to the average cost of 13 months of PAL (EUR 35,000). The second-largest contributor to total costs was hospitalizations, averaging about EUR 2,000 per patient (note that only about 50% had any hospitalizations and about 40% were followed up for more than 4 years), followed by physician visits, and injections for ovarian suppression. In the ET arm, the cost distributions were fairly similar with the exception of PAL and physician visits—patients in PAL+ET arm spent, on average, EUR 500 more on physician visits over the FU period

**Table 2:** Quality-adjusted life years and costs.

Population	All countries			Germany		
Arm	PAL + ET (*N* = 631)	ET (*N* = 619)	*p*-value	PAL + ET (*N* = 218)	ET (*N* = 214)	*p*-value
Quality of life, n						
FU, years^a^	2.82 ± 1.39	2.74 ± 1.3	0.152	3.03 ± 1.48	2.69 ± 1.44	0.012
Missing^b^ QALYs, %	42.1	42.9	0.042	37.3	42.1	0.192
Baseline utility	0.90 ± 0.13	0.89 ± 0.14	0.205	0.91 ± 0.12	0.90 ± 0.12	0.464
Total QALYs	2.50 ± 1.31	2.42 ± 1.22	0.188	2.65 ± 1.40	2.39 ± 1.33	0.054
Total discounted QALYs	2.34 ± 1.20	2.27 ± 1.12	0.188	2.47 ± 1.28	2.24 ± 1.22	0.055
Costs, EUR						
FU, years^a^	3.38 ± 1.16	3.28 ± 1.00	0.060	3.46 ± 1.30	3.29 ± 1.00	0.111
Missing^b^ costs, %	33.6	34.8	0.353	30.7	32.7	0.617
PAL	33,193 ± 9,921	279 ± 3,138	<0.001	33,233 ± 9,812	175 ± 2,557	<0.001
Hospitalization	2,272 ± 7,450	2,302 ± 6,605	0.706	3,044 ± 5,198	4,048 ± 7,508	0.661
Screening	374 ± 437	338 ± 352	0.474	334 ± 315	323 ± 320	0.386
Physical examinations and specialist visits^c^	1,912 ± 805	1,457 ± 574	<0.001	1,947 ± 885	1,454 ± 674	<0.001
Targeted therapy	1,516 ± 6,348	2,216 ± 8,177	0.135	1,925 ± 7,261	3,125 ± 10,366	0.156
Hormone therapy	305 ± 225	309 ± 244	0.725	299 ± 246	320 ± 285	0.752
Ovarian suppression^d^	1,628 ± 4,482	1,701 ± 4,490	0.398	716 ± 2,370	793 ± 2,587	0.221
Radiotherapy	285 ± 1,415	315 ± 1,541	0.915	389 ± 1,593	535 ± 2,162	0.659
Chemotherapy	33 ± 114	33 ± 121	0.225	33 ± 100	50 ± 152	0.893
Mental health or physiotherapy	322 ± 3,840	151 ± 965	0.839	872 ± 6,469	357 ± 1,368	0.682
Total costs	41,841 ± 16,384	9,102 ± 13,145	<0.001	42,792 ± 17,050	11,180 ± 15,281	<0.001
Total discounted costs	40,237 ± 15,392	8,510 ± 12,253	<0.001	41,137 ± 15,872	10,490 ± 14,337	<0.001

Continuous variables are summarized, with a mean ± SD. Significance of differences in sample characteristics between the arms were assessed with Fisher’s exact test for binary, continuity-corrected chi-square test for categorical and Wilcoxon test for continuous parameters. ^a^FU refers to the number of years between patient entry date and last reported outcome; ^b^Missing describes the average fraction of patient-year records missing per patient within the 6-year FU period (includes both attrition and missing due to administrative censoring); ^c^Excluding visits related to administration of ovarian suppression, including examinations by physicians, referral, and follow-up visits related to screenings and hospitalizations; ^d^Includes goserelin or other luteinizing hormone-releasing hormone (LHRH) injections and not surgery or radiotherapy that are captured under the respective event types.

ET, endocrine therapy; FU, follow-up; PAL, palbociclib; QALYs, quality-adjusted life years.

### Regression-adjusted results

The mean yearly differences in outcomes between study arms were cumulated in **Table 3** to show the total health-economic effects of PAL throughout the trial FU. Unadjusted effects of PAL on QALYs were marginal during the active treatment phase and increased, favoring PAL, in later years, leading to a sizable and statistically significant effect over years 1–4 (see also **Tables A15** and **A16** in **Appendix A3** for yearly incremental differences). Regression-adjusted estimates of PAL impacts on QALYs were not significant and less favorable than the descriptive result, while the upward time trend was numerically maintained. The cumulative effect over FU years 1–4 added up to 0.09 QALYs gained and was marginally significant. Impacts on costs aligned with the descriptive result, with the bulk of incremental costs accrued in the first year; these increased only marginally throughout FU. The estimated ICER was about EUR 380,000 per QALY gained, which is nearly double the unadjusted ratio, consistent with differences in effectiveness.

**Table 3:** Cumulative incremental QALYs, costs, and cost-effectiveness ratios by year of FU without and with regression adjustment.

FU, years	Incremental QALYs, *n*	*p*-value	Incremental costs, EUR	*p*-value	ICER, EUR
Unadjusted					
1	0.003 (−0.011; 0.017)	0.668	31,422 (30,632, 32,211)	<0.001	10,248,892
1-2	0.021 (−0.010; 0.053)	0.178	32,884 (31,817; 33,950)	<0.001	1,529,645
1-3	0.064 (0.003; 0.124)	0.040	32,995 (31,606; 34,384)	<0.001	517,925
1-4	0.160 (0.041; 0.280)	0.009	33,636 (31,892; 35,380)	<0.001	209,934
Regression-adjusted					
1	0.000 (−0.012; 0.013)	0.959	31,441 (30,658; 32,224)	<0.001	93,371,819
1-2	0.013 (−0.019; 0.045)	0.437	32,863 (31,799; 33,926)	<0.001	2,579,213
1-3	0.049 (−0.008; 0.107)	0.094	32,865 (31,490; 34,239)	<0.001	667,611
1-4	0.088 (−0.001; 0.177)	0.054	33,336 (31,640; 35,033)	<0.001	380,001

The table shows mean and 95% confidence interval for cumulative incremental impacts of PAL on QALYs and costs over the respective years of FU. The unadjusted estimates were obtained by summing the incremental mean differences between FU years. Regression-adjusted estimates were obtained by summing the average marginal effects of PAL predicted for each year of FU from mixed-level linear models estimated on the full study population including data from all countries; see text for details. Data were censored to include patients who were present or dead at the end of each yearly FU. The total number of patient-year records used in the estimation was 2,987 for QALYs and 3,576 for costs. Unadjusted and regression-adjusted incremental impacts by arm and year of FU are reported in Tables A15 and A16 and mean totals by year in **Figure A7** in **Appendix A3**.

FU, follow-up; ICER, incremental cost-effectiveness ratio; QALYs, quality-adjusted life years.

### Uncertainty and scenario analysis

**Figure 1:**
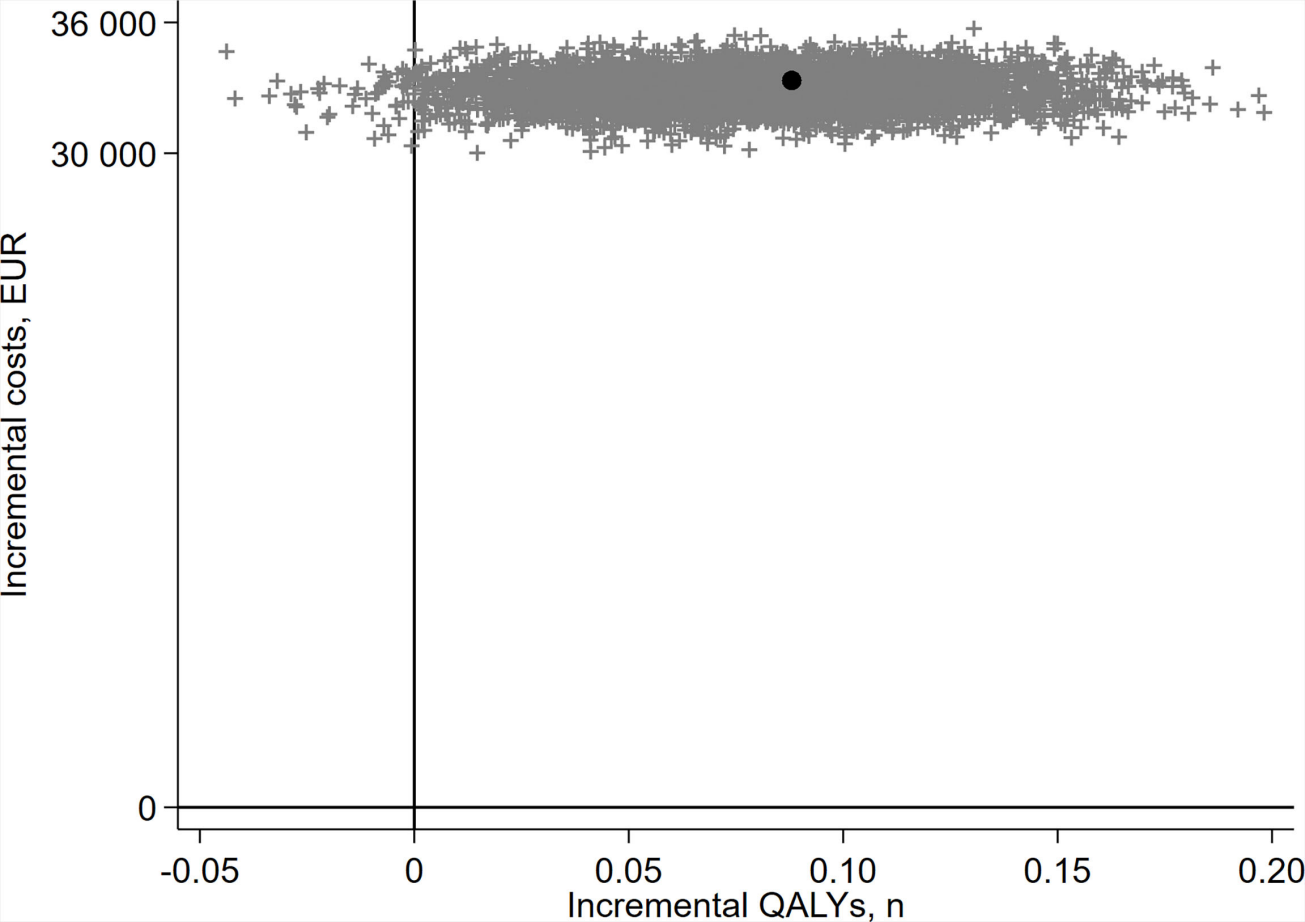
Bootstrap of regression-adjusted cumulative incremental outcomes plotted on the cost-effectiveness plane, 4 years of FU. Each dot represents a bootstrap replication (out of 5,000) of the cumulative incremental outcomes based on regression-adjusted results estimated at 4 years of FU. The black dot corresponds to the mean incremental QALYs of 0.088 and incremental costs of EUR 33,336 as reported in **Table 4** above.

**Figure 1** presents the probabilistic distribution of regression-adjusted incremental outcomes cumulated over years 1–4 from a bootstrap resampling with 5,000 replications. The plot highlights the great extent to which the uncertainty in the cost-effectiveness of PAL was driven by uncertainties on its effects on QALYs.

Key scenarios, testing the robustness of the main result, are presented in **Table 4** (see **Appendix A4** for the full set). Analyses restricted to patients recruited in Germany (scenario 1) yielded a lower point estimate on QALYs with no statistical significance and an estimated ICER exceeding EUR 1,600,000. Otherwise, effects on QALYs were marginally significant across scenarios evaluated [positive in all but the complete case analysis (scenario 16)] and fairly closely clustered around the main result. The highest incremental gains, favoring PAL, were estimated in patients who received at least 80% of PAL doses, patients over 50, patients with ypN equal to 2 or 3, and patients with a risk score ≥ 3 (scenarios 3, 8, 6, and 12). Moreover, strong positive effects of PAL were estimated when data from year 5, based on less than 9% of patients (those enrolled early enough to reach this FU time point and not censored), were included (scenario 15). The estimated incremental gains doubled between years 4 and 5, leading to a total of 0.201 QALYs gained and an ICER of EUR 167,905 over 5 years of FU. Estimates based on multiply imputed data were similarly marginally positive and quantitatively comparable to the base-case results when using MMRM (scenarios 17–19). The incremental costs ranged between EUR 31,178 and 35,974. The largest incremental costs were estimated in patients who received at least 80% of PAL doses, while the lowest—indicating greater cost savings from PAL—were in patients with Ki-67 > 15%. The ICERs were mostly above EUR 300,000 and moved in a predictable pattern with changes in effectiveness. The overall range across scenarios was between EUR 167,905 and 1,603,238 per QALY gained.

**Table 4:** Scenario analyses: regression-adjusted, 4 years of FU.

No	Rationale	Scenario	Incremental QALYs, n	*p*-value	Incremental costs, EUR	*p*-value	ICER
0		Base case	0.088 (−0.001; 0.177)	0.054	33,336 (31,640; 35,033)	<0.001	378,818
1	Heterogeneity	Patients recruited in Germany	0.021 (−0.141; 0.184)	0.797	33,668 (30,308; 37,028)	<0.001	1,603,238
2	Population	Per protocol population^*^	0.097 (0.007; 0.186)	0.035	33,381 (31,618; 35,144)	<0.001	344,134
3	Population	Patients randomized and treated	0.086 (−0.003; 0.174)	0.058	33,904 (32,240; 35,568)	<0.001	394,233
4	Population	Patients who received 80% of PAL doses	0.174 (0.087; 0.261)	<0.001	35,974 (34,392; 37,556)	<0.001	206,747
5	Risk factor	Patients with ypN 0-1	0.084 (−0.035; 0.203)	0.167	32,542 (30,203; 34,881)	<0.001	387,405
6	Risk factor	Patients with ypN 2-3	0.104 (−0.029; 0.237)	0.126	34,071 (31,521; 36,620)	<0.001	327,606
7	Risk factor	Age ≤ 50 years	0.034 (−0.086; 0.155)	0.579	34,672 (32,267; 37,076)	<0.001	1,019,765
8	Risk factor	Age > 50 years	0.157 (0.024; 0.289)	0.020	31,822 (29,468; 34,176)	<0.001	202,688
9	Risk factor	Patients with Ki-67 ≤ 15%	0.088 (−0.003; 0.179)	0.059	33,873 (31,839; 35,907)	<0.001	384,920
10	Risk factor	Patients with Ki-67 > 15%	0.078 (−0.134; 0.290)	0.472	31,335 (28,061; 34,10)	<0.001	401,731
11	Risk factor	Patients with CPS-EG score 2 and ypN+	0.062 (−0.065; 0.188)	0.341	33,415 (30,533; 36,297)	<0.001	538,952
12	Risk factor	Patients with CPS-EG score ≥ 3	0.094 (−0.030; 0.219)	0.138	33,401 (31,266; 35,535)	<0.001	355,330
13	Data limitations	Excluded non-breast-cancer hospitalizations	0.088 (−0.001; 0.177)	0.054	33,178 (31,571; 34,786)	<0.001	377,023
14	Data limitations	Included imputed expenditure in the year of death	0.088 (−0.001; 0.177)	0.054	33,293 (31,591; 34,995)	<0.001	378,330
15	Data limitations	Include data through year 5	0.201 (0.069; 0.332)	0.003	33,749 (31,450; 36,048)	<0.001	167,905
16	Missing values	Complete case analysis	−0.027 (−0.079; 0.025)	0.311	34,672 (32,745; 36,598)	<0.001	Detrimental
17	Missing values	MICE, OLS	0.103 (0.015; 0.191)	0.022	33,287 (31,655; 34,919)	<0.001	323,175
18	Correlation between outcomes	MICE, SUR	0.096 (0.000; 0.192)	0.051	35,070 (31,502; 38,638)	<0.001	365,313
19	Skewed outcomes	MICE, GLM	0.103 (0.015; 0.190)	0.021	33,288 (31,669; 34,907)	<0.001	323,184

The table presents the estimated regression-adjusted mean (95% confidence interval) differences between the arms in QALYs gained and costs incurred at 4 years of FU. The estimates were obtained by summing the average marginal effects of PAL predicted for each year of FU from mixed-level linear models; see text for details. Unless stated otherwise, data on QALYs and costs were censored to only include patients who were present or dead at the end of each yearly FU; item-missingness was relatively few and filled according to the algorithms detailed in the text. ^*^ See Loibl et al. (2021) (16)for exclusion of patients from per-protocol analysis. Scenarios 16–19 entailed multiple imputation with chained equations; missing values were filled following predictive mean matching (radius, five patients). Further details on MICE are in **Appendix A2**. The full set of scenarios evaluated are reported in **Table A17** in **Appendix A4**

FU, follow-up; ICER, incremental cost-effectiveness ratio; GLM, generalized linear model; OLS, ordinary least squares; PAL, palbociclib; SUR, seemingly unrelated regressions; QALYs, quality-adjusted life years.

## Discussion

### Key findings

We analyzed HRQoL and resource use data from the PENELOPE-B trial to estimate from the perspective of the German health statutory insurance the incremental effectiveness, expressed in QALYs, and costs of PAL added to standard-of-care ET in women with early breast cancer and at a high risk of relapse. Our primary result, regression-adjusted for stratification, missing data, and any residual imbalances at randomization, showed positive and marginally significant impacts of PAL on HRQoL at 4 years of FU. These impacts did not translate to differential care-seeking or cost savings, with nearly the full cost of PAL passed on to the system. The ICER was estimated at about EUR 380,000 per QALY gained, implying that PAL was not cost-effective compared to the standard-of-care ET at conventional willingness-to-pay thresholds.

We additionally observed that the effects of PAL on QALYs increased over time. We estimated relatively large and significant QALY gains in year 5, which led to an overall positive and significant cumulative effect over 5 years. This result should be interpreted with caution since few patients were followed this long. While PAL remained not cost-effective, more mature data on the effects of PAL beyond year 4, from PENELOPE-B or other trials, would be required to clarify the longer-term effects and, potentially, also the health-economic properties of PAL. We estimated numerically greater effects when averaged over all participating countries compared to Germany, suggesting that PAL+ET might be more effective in some settings. The differences in the magnitude and statistical significance compared to the full population were consistent with differences in patient characteristics and loss in power due to reduced sample size.

### Policy implications and significance

To date, the economic analyses of PAL and other CDKi primarily focused on advanced and metastatic patients (42–47); these studies relied on data from several CDKi trials (48–52) to extrapolate impacts of CDKi to lifetime horizons in cohorts of patients. Overall, these model-based analyses estimated the incremental effectiveness of PAL compared to ET (most often with letrozole) between 0.32 and 1.39 QALYs gained with resulting ICERs, sensitive to price assumptions, between USD 150,000 and 800,000 per QALY (42–47). To our knowledge, this is the first study that evaluated the health-economic properties of PAL in early breast cancer patients. Considered together with the evidence of no clinical benefit shown by Loibl et al. (16), our findings do not support adding PAL to the standard-of-care ET in the adjuvant setting in high-risk patients in Germany.

### Limitations

The analysis is subject to several limitations that may have impacted our results. These primarily stem from difficulties of collecting comprehensive resource use data alongside a clinical trial (53). Data collection forms were designed to minimize patient burden leading to some challenges in interpreting and valuing resource use (see **Supplementary Materials 1**). While these issues may have affected the level of costs, potentially biasing our estimates downward (54), the impact on incremental costs was likely minor since both clinical events and care episodes were well-balanced between the trial arms. Moreover, the costs of the care episodes most affected were relatively small compared to the initial costs of PAL treatment. These considerations were further supported with scenario analyses (**Table A17** in **Appendix A4**, scenarios 13–23).

We carefully considered different sources of missing values in our data and applied appropriate strategies for valid inference. First, administrative censoring did not introduce bias but limited power to infer the impact of PAL over the maximum trial FU. Item-level missingness, to the extent that we could identify it, was relatively limited, leading us to adopt some *ad-hoc* solutions. We interpolated between FU points to fill in missing utility values and borrowed information from unaffected patients on types and quantities of resources used, which might have introduced some bias. The bigger challenge was dealing with attrition, which increased over the trial FU for both QALYs and costs. Our modeling strategy—MMRM—has been shown to be valid under MAR provided the random-effects structure was correctly specified (38). We used multiple imputation to allow for a more flexible correlation structure in patient random effects over time (38, 39). The estimated effects and their significance aligned well between the two methods.

Finally, we opted to model the outcomes with linear models despite both QALYs and costs being highly (left- and right-) skewed, yielding biased estimates of the mean. The main advantage of this functional form is the ease of interpretation; i.e., the estimated coefficients are directly interpretable as incremental impacts and can also be directly compared to descriptive means. Specifications using GLM with gamma log-link family following MICE that appropriately captured skewness in the data produced estimates that were nearly identical to our main specification (equal for impact on QALYs and higher for the costs with wider confidence intervals), suggesting that the linear models adequately captured the incremental differences between the arms.

## Data availability statement

All relevant data are within this paper and its supporting information files. The data underlying the clinical results presented in the study are available from the German Breast Group. Some restrictions apply because of the confidentiality of patient data. Since these data are derived from a prospective clinical trial with ongoing follow-up collection, there are legal and ethical restrictions to sharing sensitive patient-related data publicly. Interested groups may use the Cooperation Proposal Form at https://www.gbg.de/en/research/trafo.php. Data can be requested in the context of a translational research project by sending the form to trafo@gbg.de. Translational research proposals are approved by the GBG scientific boards.

## Ethics statement

This study was reviewed and approved by Country ethics committees or institutional review boards. The patients/participants provided their written informed consent to participate in this study.

## Author contributions

KG developed the analytical methodology, sourced unit costs and other inputs for the calculation of QALYs and costs, conducted the analysis, and wrote the first draft. SL acquired funding, designed the clinical study, and led data collection and analysis of clinical outcomes. PS sourced unit costs and other inputs for the calculation of QALYs and costs, and supported the analysis. FM, MM, MU, HRB, S-BK, HDB, NM, KAG, JG-S, CK, TR, MT, HR, MG, and AM collected the data. NB led the analysis of clinical outcomes. MS acquired funding, designed the health-economic study, supported the analysis, and revised the manuscript. All authors contributed to the article and approved the submitted version.

## Funding

This work was supported by the German Breast Group, which received funding from Pfizer. Pfizer supported the conduct of the trial financially and provided the drug. Pfizer was not involved in the design of the health-economic study, collection, analysis, or interpretation of data; in the writing of the report; or in the decision to submit the article for publication.

## Acknowledgments

We thank Daniel Droeschel for providing an overview of market reimbursement and healthcare financing within the German healthcare system and helping source unit costs, Sabine Seiler and Jenny Furlanetto for providing expert guidance on clinical management and patient pathways in early breast cancer, Christiane Praetor for clarifying data collection and data processing within PENELOPE-B trial and related documentation, and Michaela Barbier and Judith Lupatch for helpful discussions on methods for dealing with missing data in randomized controlled trials. The contributors are not in any way responsible for the content of this manuscript. The responsibility for the content is with the authors.

## Conflict of interest

KG received research funding from Novartis paid to the institution. SL received research funding from Abbvie, AstraZeneca, Celgene, Daiichi Sankyo, Immunomedics/Gilead, Novartis, and Pfizer; royalties (Digital Ki67 Evaluator) and honorarium from Abbvie, Amgen, Bayer, BMS, Celgene, Eirgenix, GSK, Lilly, Merck, AstraZeneca, Pierre Fabre, Prime/Medscape, Daiicho Sankyo, Novartis, Pfizer, Immunomedics/Gilead, Puma, Seagen, and Samsung; and reported patents (EP14153692.0, EP21152186.9, EP15702464.7, EP19808852.8, Digital Ki67 Evaluator) all paid to the institution. PS received research funding from Novartis paid to the institution. FM received research funding from Roche, Novartis, AstraZeneca, GSK/Tesaro, MED, Clovis, Vaccibody, Gilead Sciences, and Eisai, and consulting fees from Vacibody all paid to the institution; received personal consulting fees and honorarium from AstraZeneca, Clovis, GSK/Tesaro, Eli Lilly, Novartis, Pfizer, Roche, Eisai, GenomicHealth, Myriad Genetics, PharmaMar, MSD, Immunomedis/Gilead, Pierre-Fabre, AGENDIA, and Seattle Genetics, and support for meetings and travel from Pfizer, Roche, and AstraZeneca; and participated in Advisory Boards for Palles and Amgen. MU received consulting fees from Abbvie, Astra Zeneca, Amgen, Celgene, Daiichi Sankyo, Eisai, Gilead, Lilly, Molecular Health, MSD Merck, Mylan, Novartis, Pierre Fabre, Pfizer, Roche, and Seagen, and honorarium from Amgen, Astra Zeneca, BMS, Celgene, Daiichi Sankyo, Gilead, GSK, Lilly, Mundipharma, Novartis, Pierre Fabre, Pfizer, Roche, Sanofi Aventis, and Seagen all paid to the institution. HRB received personal consulting fees from AstraZeneca, and received support for attending meetings and travel from Pfizer, Daiichi Sankyo, and Roche. S-BK received research funding from Novartis, Sanofi-Aventis, and DongKook paid to the institution; received personal consulting fees and honorarium from Novartis, AstraZeneca, Eli Lilly, Dae Hwa, ISU Abxis, Daiichi Sankyo, and BeiGene; participated in Advisory Boards for Novartis, AstraZeneca, Eli Lilly, Dae Hwa, ISU Abxis, Daiichi Sankyo, and BeiGene; serves as a Co-Chair for ESMO Breast 2021-2022; and holds stock of Genopeaks and Neogene TC. HDB received research funding from NSABP to the institution and reported stock ownership in Pfizer. NM participated in an Advisory Board for Pfizer. KAG received research funding from Pfizer, AstraZeneca, Eli Lilly, Roche, Merck, Gilead, Novartis, Ayala, BMS, and Seagen paid to the institution; received personal consulting fees and honorarium from AstraZeneca, Eli Lilly, Pfizer, Merck, Novartis, Gilead, Seagen, and GenomicHealth; received payment from expert testimony from Genetech; and participated in Advisory Boards for Ayala and AstraZeneca. JG-S received personal consulting fees and honorarium from AstraZeneca, Gilead, Novartis, Daiichi Sankyo, Eli Lilly, Exact Sciences, Seagen, Sanofi, EISAI, MSD, and Celgene. CK received research funding from Health Research Board and Mater Foundation paid to the institution; received personal consulting fees and honorarium from Exact Sciences and Daiichi Sankyo; received support for attending meetings and travel from Daiichi Sankyo and Roche; and participated in Advisory Board for Daiichi Sankyo. TR received personal consulting fees and honorarium from Pfizer. MT received research funding from Chugai, Takeda, Pfizer, Kyowa-Kirin, Taiho, JBCRG assoc., KBCRN assoc., Eisai, Eli Lilly, Daiichi Sankyo, AstraZeneca, Astellas, Shimadzu, Yakult, Nippon Kayaku, AFI technology, Luxonus, Shionogi, and GL Science paid to the institution; received honorarium from Chugai, Takeda, Pfizer, Kyowa-Kirin, Taiho, Eisai, Daiichi Sankyo, AstraZeneca, Eli Lilly, MSD, Exact Science, Novartis, Shimadzu, Yakult, Nippon Kayaku, and Devicore Medical Japan; and participated in Advisory Boards for Kyowa-Kirin, Daiichi Sankyo, Eli Lilly, BMS, Athenex Oncology, Bertis, Terumo, and Kansai Medical Net. HSR received research funding (study materials only) from Pfizer, Merck, Novartis, Eli Lilly, Roche, Daiichi Sankyo, Seattle Genetics, Macrogenics, Sermonix, Boehringer Ingelheim, Polyphor, AstraZeneca, Ayala, and Gilead paid to the institution; and received personal consulting fees and honorarium from Napo, Puma, Mylan, and Samsung. MG received personal consulting fees and honorarium from AstraZeneca, Eli Lilly, Daiichi Sankyo, Amgen, Veracyte, Novartis, Pierre Fabre, MSD, and Life Brain; received payment for expert testimony from Eli Lilly; and reported an immediate family member employed by Sandoz. AM received personal consulting fees and honoraria from Pfizer. MS received research funding from AbbVie, Biogen, Bristol Myers Squibb, Merck Sharpe and Dohme, Mundipharma, Novartis, and Roche paid to the institution; and received personal consulting fees from BMS and Sandoz.

The remaining authors declare that the research was conducted in the absence of any commercial or financial relationships that could be construed as a potential conflict of interest.

## Publisher’s note

All claims expressed in this article are solely those of the authors and do not necessarily represent those of their affiliated organizations, or those of the publisher, the editors and the reviewers. Any product that may be evaluated in this article, or claim that may be made by its manufacturer, is not guaranteed or endorsed by the publisher.
