# Supplementary material for: Cost-effectiveness of palbociclib in early breast cancer patients with a high risk of relapse: Results from the PENELOPE-B trial
Source: Front Oncol. 2022 Sep 5;12:886831. doi: 10.3389/fonc.2022.886831 (PMC9484462; doi:10.3389/fonc.2022.886831)
Supplement: Supplementary file 1 [file DataSheet_1.docx]

**Supplementary Material**

**Cost-effectiveness of *palbociclib* in early breast cancer patients with a high risk of relapse: results from the PENELOPE-B trial**

Katya Galactionova^1*^, Sibylle Loibl^2^, Paola Salari^1^, Frederik Marmé^3^, Miguel Martin^4^, Michael Untch^5^, Hervé R. Bonnefoi^6^, Sung-Bae Kim^7^, Harry D. Bear^8^, Nicole McCarthy^9,10^, Karen A. Gelmon^11^, José A. García-Sáenz^12,13^, Catherine M. Kelly^14^, Toralf Reimer^15^, Masakazu Toi1^6^, Hope S. Rugo^17^, Michael Gnant^18^, Andreas Makris^19^, Nicole Burchardi^2^, and Matthias Schwenkglenks^1^

1 Institute of Pharmaceutical Medicine (ECPM), University of Basel, Basel, Switzerland

2 German Breast Group, Neu-Isenburg, Germany

3 Medical Faculty Mannheim, Heidelberg University, University Hospital Mannheim, Mannheim, Germany

4 Instituto de Investigacion Sanitaria Gregorio Marañon, CIBERONC, Universidad Complutense, Madrid, Spain and Spanish Breast Cancer Group, GEICAM, Madrid, Spain

5 Helios Kliniken Berlin-Buch, Berlin, Germany

6 Institut Bergonié and Université de Bordeaux INSERM U916, Bordeaux, France

7 Asan Medical Center, University of Ulsan College of Medicine, Seoul, Korea

8 Division of Surgical Oncology, Massey Cancer Center, Virginia Commonwealth University, VCU Health, Richmond, VA, USA

9 Australia and New Zealand Breast Cancer Trials Group, Newcastle, Australia

10 University of Queensland, Queensland, Australia

11 BC Cancer, Vancouver, British Columbia, Canada

12 Instituto de Investigación Sanitaria del Hospital Clinico San Carlos (IdISSC), Madrid, Spain

13 GEICAM, Madrid, Spain

14 Mater Private Hospital, Cancer Trials Ireland, Dublin, Ireland

15 Department of Obstetrics and Gynecology, University of Rostock, Rostock, Germany

16 Breast Surgery, Graduate School of Medicine, Kyoto University, Kyoto, Japan

17 University of California San Francisco Comprehensive Cancer Center, San Francisco, CA, USA

18 Comprehensive Cancer Center, Medical University of Vienna, Vienna, Austria

19 Institute of Cancer Research, London, UK

* Corresponding author:

Katya Galactionova
e.galactionova@unibas.ch

Contents

[Supplementary File 1. Recording of resource use in the PENELOPE-B trial and coding routines implemented to facilitate costing 4](#_Toc96956306)

[Supplementary Table 1. Frequency of data collection by type of resource use costed 4](#_Toc96956307)

[Supplementary Table 2. Text snippets used to recode of free-text entries into screenings and respective frequencies (number of records) 6](#_Toc96956308)

[Supplementary Table 3. Text snippets used to recode of free-text entries into types of hospitalizations and respective frequencies (number of records) 8](#_Toc96956309)

[Supplementary Table 4. Rules for filling in missing values 10](#_Toc96956310)

[Supplementary Table 5. Unit costs and sources by service type (EUR, 2020) 13](#_Toc96956311)

[Supplementary Table 6. Number of missings by outcome, arm and year 15](#_Toc96956312)

[Supplementary Table 7. Missing data patterns 16](#_Toc96956313)

[Supplementary Table 8. Associations between missingness and baseline characteristics 17](#_Toc96956314)

[Supplementary Table 9. Associations between missingness and outcomes 19](#_Toc96956315)

[Supplementary Table 10. Testing sample heterogeneity: total QALYs, and total costs at average FU by country and arm 20](#_Toc96956316)

[Supplementary Table 11. Model selection: QALYs 21](#_Toc96956317)

[Supplementary Table 12. Model selection: costs 23](#_Toc96956318)

[Supplementary File 2. Technical details on MICE implementation and diagnostics 25](#_Toc96956319)

[Supplementary Material File 3. Supplementary results 32](#_Toc96956320)

[Supplementary Table 13. Patient baseline characteristics 32](#_Toc96956321)

[Supplementary Table 14. Distribution of average cost per patient by type of care and t, EURs 34](#_Toc96956322)

[Supplementary Figure 7. Average annual cost per patient in the first year of FU by treatment arm, EURs 36](#_Toc96956323)

[Supplementary Figure 8. Distribution of average total cost per patient by type of care and FU year, EURs 37](#_Toc96956324)

[Supplementary Figure 9. Average cost by type of care per patient and t 38](#_Toc96956325)

[Supplementary Figure 10. Average cost by type of care per patient and t conditional on any having any care 39](#_Toc96956326)

[Supplementary Figure 11. Average number of visits/ pills/ inpatient days by type of care per patient and t 40](#_Toc96956327)

[Supplementary Figure 12. Average number of visits/ pills/ inpatient days by type of care per patient and t conditional on any having any care 41](#_Toc96956328)

[Supplementary Table 15. Unadjusted incremental quality-adjusted life years, costs and ICER by year of FU 42](#_Toc96956329)

[Supplementary Figure 13. Regression-adjusted predicted quality-adjusted life years and costs by year of FU 43](#_Toc96956330)

[Supplementary Table 16. Regression-adjusted incremental quality-adjusted life years, costs and ICER by year of FU 44](#_Toc96956331)

[Supplementary File 4. Scenario analyses 45](#_Toc96956332)

[Supplementary Table 17. Scenario analyses: regression-adjusted cumulative incremental outcomes and cost-effectiveness ratios at 4 years of FU 46](#_Toc96956333)

[Supplementary File 5. CHEERS checklist 48](#_Toc96956334)

# Supplementary File 1. Recording of resource use in the PENELOPE-B trial and coding routines implemented to facilitate costing

Medical resource use recorded in the trial covered all care episodes including those related to causes other than breast cancer. Care episodes occurring at the enrolling and treating medical centers were transferred from the patients' medical records. These covered diagnostic procedures and treatments received before randomization; study drug and other cancer-related drugs until end of treatment (EOT); laboratory tests, cardiac monitoring and imaging procedures performed until EOT; adverse events occurring until EOT; endocrine treatment during follow-up; diagnostic screenings during follow-up; other cancer treatments during follow-up. Care episodes occurring elsewhere were transferred from patient diaries. These served to record the intake of the study drug and care episodes (outpatient physician visits and hospitalizations) occurring until EOT and during FU. Supplementary Table 1 gives the frequency of FU for each type of care episode; multiple events were captured under FU assessment, FU other cancer treatment, relapse and AE forms.

## Supplementary Table 1. Frequency of data collection by type of resource use costed

| Type | Event | Frequency |
| --- | --- | --- |
| PAL | PAL | Baseline, Cycles 1-13, EOT |
| ET | ET | Baseline, Cycles 1-13, EOT |
| Hormone therapy | Ovarian suppression | Baseline, Cycles 1-13, EOT |
| Physician visits | Cardiac monitoring | Baseline, Cycle 1 and as indicated |
| Physician visits | Physician examinations | Baseline, Cycles 1,2,3,4,5,6,7,9,11,13, EOT |
| Physician visits | Outpatient physician visits | Baseline, Cycles 1,3,5,7,9,11, EOT |
| Diagnostic screenings | Imaging | Cycle 7 and as indicated |
| Multiple | FU assessment | Bi-annually in FU years 1-4 and once a year thereafter |
| Multiple | FU other cancer treatments | Bi-annually in FU years 1-4 and once a year thereafter |
| Multiple | Relapse | Baseline, Cycles 1-13, EOT, bi-annually in FU years 1-3 and once a year thereafter |
| Hospitalizations | Hospitalizations | Baseline, Cycles 1,3,5,7,9,11, EOT, bi-annually in FU years 1-3 and once a year thereafter |
| Multiple | AE | Cycles 1-13, EOT, bi-annually in FU years 1-4 and once a year thereafter |

Information on the medical resource use generally allowed characterization of care episodes with respect to the specific agent or type of care received, the number of events since last FU, and their duration facilitating costing. However, the level of detail varied by type of care episode and also throughout the trial. While PAL and ET were tracked in detail (recorded date of administration, dose, length, and reason for treatment delay or interruption) throughout the trial FU, other cancer treatments including radio- and chemotherapies were only recorded with a start and end date requiring additional assumptions on the drug, regimen, dose, and adherence. Information collected on diagnostic screenings, concomitant medications, hospitalization, and physician visits covered date of the visit or start and end dates for drugs and hospitalizations and dosage (if applicable); all identifying information on the type of assessment and the clinical indication was entered as free-text. Finally, information on adverse events covered date, severity of the episode (based on CTC grade), and an indicator for whether or not the event was related to the study drug. Related care episodes were not recorded.

Coding routines were developed to mine free-text entries in order to map care episodes into line items that could be consistently costed. Entries on diagnostic screenings were grouped into 9 line items; if a text entry matched multiple assessments the record was duplicated accordingly (see Supplementary Table 2 for text snippets used and the corresponding frequencies). For hospitalization, we adopted the nomenclature developed by US Agency for Healthcare Research and Quality [1] grouping all hospitalizations into minor surgery, resection, mastectomy, reconstruction surgery, other surgeries, other non-surgical hospitalizations, and hospitalizations related to injury (see Supplementary Table 3 for text snippets used and the corresponding frequencies). We ignored physician visits recorded in patients’ diaries since it was not possible to distinguish from the free-text entries physician consultation visits from screenings and hospitalizations recorded elsewhere. Instead, we imputed physician visits based on other care episodes (See Supplementary Table 4). Physician visits entered by trial staff from patient medical records were retained as reported. Concomitant medications were not considered due to difficulties in systematically identifying treatments from free-text entries. Lastly, we excluded care episodes with minor cost implications including laboratory blood tests until EOT and minor adverse events (CTC grade <3).

## Supplementary Table 2. Text snippets used to recode of free-text entries into screenings and respective frequencies (number of records)

| No | Type | Line item | Snippet | Fraction | N |
| --- | --- | --- | --- | --- | --- |
| 1 | Screening | Computed tomography | tomog ct-scan ct- scan ct(contrast) ct(body) ct- chest ct-thorax chest scan “pelvis scan" "thorax scan" "scanner" "tac-tap" "tac scan" "cat scan" "bilan radiosenologique" "pelvic scan" "tdm tap" "coronary artery study" | 0.094 | 546 |
| 2 | Screening | PET | pet "tep scan" "positron emission tomography" "pet scanner" | 0.016 | 96 |
| 3 | Screening | Skeletal scintigraphy | szintigarphie szinti sciniti scinti nuclear "bone radio imaging" "gamma scan imaging" "bone scan" "bine scan" "bone-scan" bonescan "bone spect" "gamma scan" | 0.036 | 209 |
| 4 | Screening | Bone densitometry | densitome dexa dxa density densiome | 0.010 | 56 |
| 5 | Screening | Ultrasound | ultrasound ultra sound breast-us sono "breast us" "us breast" mmgus "u/s" "u/a" echography ecography echo ecog mmgus doppler | 0.233 | 1359 |
| 6 | Screening | Mammogram | mammog mamogram mamamogr mamogr mommo mmg mammorg mammagr mamaogr ammography mammpgra tomosyn "radiological examination" | 0.263 | 1534 |
| 7 | Screening | MRI | mri rmi magnetic rmn mrt "mrs mammae" imaging "breast rm" "breast rmi" "breast rmn" | 0.037 | 213 |
| 8 | Hospitalization | Minor surg/ Biopsy | biops "fine needle" puncture breastbone aspiration histological histopathology "ebus-tbna" "right supraclavicular fna" vabb resection retromamillary vats cholecystectomy laparotomy adnexectomy | 0.019 | 112 |
| 9 | Radiotherapy | Radiation ther | radiotherapy | 0.000 | 1 |
| 10 | Screening | X-ray | "x-ray" xr "x ray" xray "chest pa" radiography apct "x-p" | 0.027 | 155 |
| 11 | Physician visit | Gynaecologist | exam physical "kÃ¶rperliche untersuchung" phyex fisic control obgy gynecology gynÃ¤kology gynecologe gynaecological gynaecologist "vaginal bleeding" eye palpation inspection clinical exploration vital visit visite clinic outpatients assessment "gynecological prevention" oncology onkologie "check-up" "check up" "no breast lumps" "no palpable masses" "vascularization increase" "plastic surgeon" amenorrhea exploration hpi "overall health" ophthalmologist "no relapse signs" "breast, chest, abdominal and calves" "checked in urology" "history of present illness" "malaise/fatigue (grade 1), joint pain" ent onkology follow-up aftercare "after care" routine aftertreatment "follow up" f/u "fup visit" fup "3 month fu" "inject denusom" palpated oncologist "family medicine" "general practitioner" "mastectomy site ok" "patient seen by" "no palpable nodes" conversation information consult review documents revision "talk about" "referral request" prescri explanation planning phone call "contacted patient" "plan to use faslodex and xgeva" | 0.339 | 1976 |
| 12 | Physician visit | Pap smear | smear "cervical cytology" | 0.001 | 4 |
| 13 | Hospitalization | Breast reconstruction | reconstr rekonstruktion mammoplasty prosthesis "plastic surgery" recostruction lipofil implant | 0.001 | 3 |
| 14 | Laboratory | Laboratory hema | blood hematology heamocult serum "tumor markers" "ca 15-3" "ca15-3" "tumor marker" laboratory | 0.009 | 52 |
| 15 | Screening | ECG | cardio ecg fevi | 0.001 | 6 |
| 16 | Screening | Endoscopic examination | endosc colonoscopy polypectomy oesophagoscopy broncho | 0.003 | 15 |
| 17 | Hospitalization | Mastectomy | mastectomy | 0.000 | 1 |

## Supplementary Table 3. Text snippets used to recode of free-text entries into types of hospitalizations and respective frequencies (number of records)

| No | Type | Line item | Snippet | Fraction | N |
| --- | --- | --- | --- | --- | --- |
| 1 | Hospitalization | Medical | relaps metast "second cancer" progression progressive recurrence metatases "breast cancer" "breast ca " "mamma ca" progress suspect "susp of lae" "fnp liver" "ductal carcinoma" "meningeosis carcinomatosa" seroma abscess "breast inflammation" mastitis heart cardiac stroke headache migraen fatigue vertigo infect "gall bladder" herpes uti wound respiratory septic lung pain pneumo influenza "pleural effusion" dyspnea dyspnoe pharyngitis pyelonephritis thrombocytopenia arthrosis "general condition" palsy syncope emergency allergy brachial burns diarrhea carcinoma dehydration deterioration diverticulitis bleeding edema fever endometriosis erysipelas cirrhosis vomiting fibromyalgia "functional deficits" gastroenteritis gastroenteritis "generalized body aches" weakness haemorrhage hernia "hospital sligo" hypercalcemia "cervical ln" hypertens hyperthyroidism hypertonia infarct ischemia erysipelas meningitis sclerosis colic viral "general health" swelling hypertension shingles wart "streptococcus sepsis" phlebitis tachycardia "swollen right big toe pad" encephalitis "carpal tunnel" thrombosis "pancreatic carcinoma" "uterus inflammation" "vaginal bleed" "hypertention cerebrale" "conspicuous ct abdomen" bleeding bleed "patient intook 5 lorazepam pills due to autolysis" "cns mets" lymphedema lympho lymphed lymphaedema lymphadema cellulitis bronchitis divertikulose thromboembolism ascites erysipel "lws syndrom" "lws syndrome" "endometrial cancer" "peritoneal carcinosis" "neutropenic sepsis" nsteacs | 0.293 | 255 |
| 2 | Hospitalization | Injury | fractur accident broken injury fall sprain "fell down stairs" "hit by car" | 0.033 | 29 |
| 3 | Hospitalization | Surgery | cutterage curettage conisation conization laprosc abrasio laparosc cholecystectomy adnectomy polyp cyst arthroscopy meniscectomy stent "stone removal" extirpation thyroidectomy "removal of thyroid gland" "knee replacement" "gastric bypass" "surgical procedure" paritidectomy lobectomy myomectomy "macular hole" "repair torn right thumb" sugery "surgical intervention" "surgical resection of the glandula" "repair torn right thumb" "surgery metastasic" histerect anexectomy hysterect "remove ovaries" adnexectomy oophorectomy oophrectomy oopherectomy salpingo ovariectomy annexectomy "removing the ovaries" ablatio "breast nodule surgery" "heart surgery" "lymphnode exstirpation" lymphadenectomy "removal lymph nodes" cath catheter port "lymph nodes exstirpation" lyphknottransplatation "op after histolog result relaps" "removal of liver metastase" thoracoscopy "tumor excision" "tah bso" operation surgery "hysteroscopy d&c" "capsule fibrosis" "flap cut of large left dorsal" | 0.245 | 213 |
| 4 | Hospitalization | Mental health | rehab depression psychosis suicidal "drug overdose" | 0.014 | 12 |
| 5 | Hospitalization | Physiotherapy and lymph drainage | "health resort" physiother rehabilitation "lymphadema therapy" "photodynamic therapy" | 0.053 | 46 |
| 6 | Hospitalization | Breast resection | mammaresection "segmental resection" resection | 0.008 | 7 |
| 7 | Hospitalization | Breast reconstruction | reconstr recunstr expander implant lipofil "breast correction" reduction capsulect diep plastic "mamma lifting" prostethis prosthesis cosmetic plasty "adjustment of right breast" "breast construction" correction rekonstruction revision lipolifting lipoffiling aesthetical "breast wound closure" mastopexy "deformity of breast rt" "reconstuction breast left site" "breast adjustment" | 0.289 | 251 |
| 8 | Hospitalization | Mastectomy | mastecto "amputation of the breast" "prophylactic surgery" mastektomy | 0.026 | 23 |

## Supplementary Table 4. Rules for filling in missing values

| Item missing | Where observed | General rule* | Key assumptions |
| --- | --- | --- | --- |
| Line item | Ovarian suppression, hospitalizations, screening, AE, other cancer treatments prior and post event | Most frequent or based on indication as informed by clinicians | - For chemotherapy assumed Capecitabine (500 mg) [2, 3] - Assumed trenantone is used as other LHRH [2, 3] - For AE related to study drug a course of antibiotics and 1 physician visit were assigned, for severe events (CTC grade 4 and reported as a severe adverse event) length of hospitalization was set to a median stay of 6 days based on a study from Spain [4] |
| Dose | PAL, ET, ovarian suppression, chemotherapy, radiotherapy | Average or based on indication as informed by clinicians | - PAL price did not depend on dosage thus was not considered - For ovarian suppression assumed dose 36 mg for goserelin/ 375 mg for trenantone during PAL active treatment phase and injections dose 108/ 1125 mg for goserelin and trenantone during the FU phase - Average dose for radiotherapy based Arnold et al [5] - For chemotherapy assumed Capecitabine (500 mg) [2, 3] |
| Quantity | Ovarian suppression, chemotherapy, radiotherapy, ET during FU, targeted therapy for recurrence or secondary malignancy, screening, physician visits, lymph drainage, physiotherapy and talk therapy, AE | Average or based on indication as informed by clinicians | - For ovarian suppression assumed monthly injections during PAL active treatment phase and injections once every three months during the FU phase matching the routine FU schedule for breast cancer patients - Assumed chemotherapy (drug in-take at home) and radiotherapy (outpatint hospital) took place 5 times per week [2, 3] - For ET assumed daily drug intake [2, 3] - For screenings, each assessment matched was assumed to take place on its own (i.e. MRI/CT would be costed as 2 separate tests) - Physician visits were imputed based on other care episodes as follows: 1 gynecologist visit was assumed per each goserelin injection, for diagnostic screenings and other assessments during the follow-up period 2 visits with a gynecologist were added (1 for the referral prior to assessment and 1 after 1 week for interpretation and diagnosis), 3 visits with a gynecologist were added for each surgery, excluding biopsy (1 week prior to surgery for referral, 1 week after the discharge and 1 month after discharge for monitoring) - The number of pills for targeted therapy was calculated assuming 21 pills per month, following PAL regimen [6] - Lymph drainage, physiotherapy and talk therapy were assumed to take place once per week [2, 3] - If the duration was deemed to be outside of the plausible range (i.e. errors in start/ end date) the calculated value was set to zero and updated with sample average - AE related to study drug a course of antibiotics and 1 physician visit were assigned, for severe events (CTC grade 4 and reported as a severe adverse event) length of hospitalization was set to a median stay of 6 days based on a study from Spain [4] |
| Date (i.e. of intake, visit) | All resource line items | Date of FU, if also missing imputed based on the average interval between the FUs in the sample |  |
| End date | ET, chemotherapy, radiotherapy, physiotherapy and mental health | Sample average | End dates outside of trial FU were censored at the last FU present |

* Where possible, averages were computed within each country, line item or resource type category

2020 German price weights (i.e. unit costs) were used to value resource use. Drug prices were based on the median listed retail price per tablet calculated from Lauer-Taxe [7] net of the manufacturer discount (7% for drugs not linked to a fixed price and 10% for drugs with a fixed amount) and the pharmacy discount (EURs (EUR) 1.77). Costs of radiotherapy per session were obtained from the literature [5]. Physician visits were costed by specialty based on the average fee per visit from Kassenärztliche Bundesvereinigung (KBV) [8]; 2018 unit costs (the most recent available at the time of the study) were inflated to 2020 prices using German GDP deflator [9]. Screenings and other diagnostic examinations (i.e. CT, MRI, mammogram), the cost of lymph drainage massage, and minor surgery (i.e. biopsy) were also obtained from KVB. Physiotherapy costs (per session) were based on costs of inpatient hospital rehabilitation from Deutsche Rentenversicherung [10]. Inpatient hospital stays, grouped as described above, were costed based on an average cost per day for each type of hospitalization derived by dividing the average cost per visit by the average length of stay from appropriately grouped DRGs in the German DRG system [11]. Hospitalizations due to severe adverse events (neutropenia with Grade 3 and 4) related to the study drug were costed based on price weights for medical, non-surgerical hospitalization based on DRG weights. See Supplementary Table 5 for unit costs and further details on derivation.

Costs were calculated per care episode by multiplying the quantity of the resource line item (i.e number of doses) with the respective price weight. Costs related to care episodes and utility scored recorded in each FU were allocated over the study time (t=0 was set to randomization): equally for costs and using linear interpolation between the reporting intervals and calendar years for quality of life measurements. These data were then aggregated and summarized in yearly intervals for purposes of the analysis.

Since missings in costs for all care episodes followed the same pattern, costs were modelled directly as a sum of all care episodes and not by type of care (i.e. PAL, physician visits, hospitalizations, etc.).

## Supplementary Table 5. Unit costs and sources by service type (EUR, 2020)

| N | Category | Service | Unit | Price  (EUR, 2020) | Source | Notes |
| --- | --- | --- | --- | --- | --- | --- |
| 1 | Targeted therapy | Ibrance | Tablet | 102.75 | Lauer-Taxe [7] | Price refers to retail price net of manufacturer discount (7%) and pharmacy discount (1.77 EUR) |
| 2 | Hormone therapy | Tamoxifen | Tablet | 0.18 | Lauer-Taxe [7] |  |
| 2 | Hormone therapy | Anastrozol | Tablet | 0.66 | Lauer-Taxe [7] |  |
| 2 | Hormone therapy | Letrozol | Tablet | 0.52 | Lauer-Taxe [7] |  |
| 2 | Hormone therapy | Exemestan | Tablet | 1.10 | Lauer-Taxe [7] |  |
| 3 | Ovarian suppression | Goserelin | Injection  (36 ml) | 130.71 | Lauer-Taxe [7] |  |
| 3 | Ovarian suppression | Other LHRH | Injection  (ml 375) | 138.60 | Lauer-Taxe [7] | Assumed to be Trenantine. Price refers to retail price net of manufacturer discount (7%) and pharmacy discount (1.77 EUR) |
| 4 | Physician visit | General Practitioner | Visit | 66.12 | KBV [8] | Price refers to weighted average charge per specialty; 2018 prices inflated to 2020 using GDP deflator |
| 4 | Physician visit | Gynaecologist | Visit | 50.28 | KBV [8] |  |
| 5 | Screening | CT | Examination | 110.63 | KBV [12] |  |
| 5 | Screening | PET | Examination | 489.58 | KBV [12] |  |
| 5 | Screening | Skeletal scintigraphy | Examination | 62.19 | KBV [12] |  |
| 5 | Screening | Bone densitometry | Examination | 16.04 | KBV [12] | Not covered by KBV, priced based on X-ray |
| 5 | Screening | Ultrasound | Examination | 16.48 | KBV [12] |  |
| 5 | Screening | Mammogram | Examination | 30.10 | KBV [12] |  |
| 5 | Screening | MRI | Examination | 220.51 | KBV [12] |  |
| 5 | Screening | X-ray | Examination | 16.04 | KBV [12] |  |
| 5 | Screening | ECG | Examination | 21.70 | KBV [12] |  |
| 5 | Screening | Endoscopic examination | Examination | 141.19 | KBV [12] |  |
| 6 | Therapy | Lymph drainage | Session | 24.39 | KBV [12] |  |
| 6 | Therapy | Mental health | Hospital day | 427.55 | G-DRG [11] | DRGs corresponding to MDC 19, 20; German DRG 2020 base rate with 2019 weights |
| 6 | Therapy | Physiotherapy | Session | 154.00 | Reha [10] | Inpatient rehabilitation clinic; 2018 EURs inflated to 2020 using GDP deflator |
| 7 | Radiation therapy | Radiation therapy | Session | 290.73 | Arnold et al [5] | Assuming on average 6 sessions per course. Price refers to weighted average charge per specialty; 2018 prices inflated to 2020 using GDP deflator |
| 8 | Chemotherapy | Chemotherapy | Tablet | 1.09 | Lauer-Taxe [7] | Assumed to be Capecitabine (500 mg). Price refers to retail price net of manufacturer discount (7%) and pharmacy discount (1.77 EUR) |
| 9 | Hospitalization | Minor surgery | Hospital day | 91.05 | KVB [12] | OPS code 1754 |
| 10 | Hospitalization | Resection | Hospital day | 958.58 | G-DRG [11] | DRG codes J07A, J07B, J07C, J25Z; German DRG 2020 base rate with 2019 weights |
| 10 | Hospitalization | Mastectomy | Hospital day | 765.27 | G-DRG [11] | DRG codes J16A, J23Z, J24B; German DRG 2020 base rate with 2019 weights |
| 10 | Hospitalization | Breast reconstruction | Hospital day | 677.20 | G-DRG [11] | DRG codes J01Z, J06Z, J10A, J10B, J12Z, J14Z, J26Z; German DRG 2020 base rate with 2019 weights |
| 10 | Hospitalization | Medical | Hospital day | 484.19 | G-DRG [11] | Includes all other non-surgical DRGs (type “M”) except mental health, resection, mastectomy, and breast reconstruction, and injury; German DRG 2020 base rate with 2019 weights |
| 10 | Hospitalization | Injury | Hospital day | 504.22 | G-DRG [11] | DRG codes X04Z, X05A, X05B, X06A, X06B, X06C, X33Z, X64Z; German DRG 2020 base rate with 2019 weights |
| 10 | Hospitalization | Surgery | Hospital day | 880.44 | G-DRG [11] | Included all surgical DRG codes (type “O”) except for the breast cancer surgeries (i.e. resection, mastectomy, and breast reconstruction); German DRG 2020 base rate with 2019 weights |

## Supplementary Table 6. Number of missings by outcome, arm and year

| Year FU | Attrition  (N (fraction)) | | | | | | Administrative censoring  (N (fraction) | | | Attrition + administrative censoring  (N (fraction) | |
| --- | --- | --- | --- | --- | --- | --- | --- | --- | --- | --- | --- |
|  | Costs | | | QALYs | | | Costs/ QALYs | | | Costs | QALYs |
|  | PAL+ET | ET | P-value | PAL+ET | ET | P-value | PAL+ET | ET | P-value |  |  |
| 1 | 40 (.06) | 42 (.07) | 0.756 | 86 (.14) | 74 (.12) | 0.382 | 0 (0) | 0 (0) | 0 | 82 (.07) | 160 (.13) |
| 2 | 63 (.1) | 64 (.1) | 0.843 | 160 (.25) | 158 (.26) | 0.932 | 0 (0) | 0 (0) | 0 | 127 (.1) | 318 (.25) |
| 3 | 127 (.2) | 154 (.25) | 0.046 | 233 (.37) | 246 (.4) | 0.295 | 0 (0) | 0 (0) | 0 | 281 (.22) | 479 (.38) |
| 4 | 121 (.19) | 126 (.2) | 0.61 | 179 (.28) | 188 (.3) | 0.426 | 51 (.08) | 52 (.08) | 0.831 | 350 (.28) | 470 (.38) |
| 5 | 49 (.08) | 61 (.1) | 0.195 | 63 (.1) | 75 (.12) | 0.225 | 294 (.47) | 290 (.47) | 0.906 | 694 (.56) | 722 (.58) |
| 6 | 13 (.02) | 10 (.02) | 0.556 | 13 (.02) | 12 (.02) | 0.881 | 531 (.84) | 511 (.83) | 0.486 | 1066 (.85) | 1067 (.85) |
| Total | 413 (.11) | 457 (.12) | 0.062 | 734 (.19) | 753 (.2) | 0.318 | 876 (.23) | 853 (.23) | 0.891 | 2600 (.35) | 3216 (.43) |

Key: ET, endocrine therapy; FU, follow-up; QALYs = Quality-Adjusted Life-Years

## Supplementary Table 7. Missing data patterns

| Outcome | Number of person records | Number of missings |
| --- | --- | --- |
| Total costs | 3680 | 2560 |
| Costs of targeted therapy | 3680 | 2560 |
| Costs of hormone therapy | 3680 | 2560 |
| Costs of ovarian suppression | 3680 | 2560 |
| Costs of physician visits | 3680 | 2560 |
| Costs of screenings | 3680 | 2560 |
| Costs of therapy | 3680 | 2560 |
| Costs of radiotherapy | 3680 | 2560 |
| Costs of chemotherapy | 3680 | 2560 |
| Costs of hospitalization | 3680 | 2560 |
| Total QALYs | 3057 | 3183 |

Pattern of missings in total costs and total QALYs

| Pattern | Number of person records |
| --- | --- |
| Neither missing | 3012 |
| Both missing | 2515 |
| Only QALYs missing | 668 |
| Only costs missing | 45 |

## Supplementary Table 8. Associations between missingness and baseline characteristics

| Dependent variable | Any missings in QALYs | Any missings in costs |
| --- | --- | --- |
| Covariates | ẞ/se | ẞ/se |
| Baseline QALY | 0.58 | 0.82 |
|  | 0.56 | 0.70 |
| PAL+ET | 1.26 | 0.66 |
|  | 0.74 | 0.47 |
| FU year 2 | 114.86*** | 46.21*** |
|  | 56.47 | 25.42 |
| FU year 3 | 3193.15*** | 4517.95*** |
|  | 2200.68 | 3655.13 |
| FU year 4 | 134063.82*** | 142123.91*** |
|  | 121096.48 | 139051.02 |
| FU year 5 | 6.17e+07*** | 2.61e+07*** |
|  | 8.44e+07 | 3.55e+07 |
| PAL+ET # FU year 2 | 0.74 | 1.30 |
|  | 0.36 | 0.79 |
| PAL+ET # FU year 3 | 0.62 | 0.94 |
|  | 0.36 | 0.64 |
| PAL+ET # FU year 4 | 0.68 | 1.51 |
|  | 0.47 | 1.15 |
| PAL+ET # FU year 5 | 0.84 | 1.87 |
|  | 0.81 | 1.75 |
| Lymph node status sur (ypN 0-1 vs ypN2-3) | 0.69 | 0.69 |
|  | 0.18 | 0.16 |
| Age at first diagnosis ≤ 50 | 2.28* | 1.58 |
|  | 0.81 | 0.49 |
| Ki-67 ≤15% | 1.44 | 0.80 |
|  | 0.42 | 0.21 |
| CPS-EG score ≥3 | 0.41** | 0.40** |
|  | 0.13 | 0.11 |
| Tumour grade G1 or G2 | 0.55 | 0.45** |
|  | 0.17 | 0.13 |
| Overall clinical response after NACT |  |  |
| CR | 3.18* | 1.42 |
|  | 1.56 | 0.60 |
| SD | 0.76 | 0.75 |
|  | 0.27 | 0.24 |
| PD | 1.45 | 4.57 |
|  | 1.75 | 4.97 |
| Breast cancer treatments pre-randomization |  |  |
| Started End before PAL | 1.27 | 1.03 |
|  | 0.54 | 0.39 |
| First ET with tamoxifen | 0.42* | 0.44** |
|  | 0.14 | 0.13 |
| Had a hysterectomy | 1.65 | 1.58 |
|  | 1.26 | 1.06 |
| Had radiotherapy | 0.15 | 0.33 |
|  | 0.18 | 0.36 |
| Had a breast reconstruction surgery | 1.74 | 1.32 |
|  | 0.61 | 0.41 |
| Number of breast surgeries |  |  |
| 2 | 1.25 | 2.33** |
|  | 0.46 | 0.76 |
| 3+ | 0.65 | 0.90 |
|  | 0.44 | 0.55 |
| Number on-going illnesses with treatment |  |  |
| 1 | 0.70 | 0.96 |
|  | 0.25 | 0.30 |
| 2 | 0.55 | 0.48 |
|  | 0.26 | 0.20 |
| 3 | 0.92 | 0.75 |
|  | 0.53 | 0.38 |
| Has a cardiac disorder | 3.67 | 2.29 |
|  | 4.24 | 2.38 |
| Has a vascular disorder | 1.54 | 1.57 |
|  | 0.63 | 0.57 |
| Has a psychiatric disorder | 0.84 | 1.28 |
|  | 0.37 | 0.50 |
| Country |  |  |
| Spain | 5.77*** | 1.65 |
|  | 2.25 | 0.55 |
| France | 15.06*** | 4.71*** |
|  | 7.72 | 2.06 |
| Austria | 1.05 | 0.89 |
|  | 0.98 | 0.74 |
| Ireland, UK | 6.43*** | 14.22*** |
|  | 4.40 | 8.97 |
| USA | 3.29*** | 6.54*** |
|  | 1.50 | 2.71 |
| Australia | 1.22 | 2.11 |
|  | 0.68 | 1.05 |
| Japan | 0.13** | 0.84 |
|  | 0.10 | 0.55 |
| S. Korea | 0.29 | 1.28 |
|  | 0.20 | 0.78 |
| var(t[id]) | 477.24 | 144.69 |
|  | 555.48 | 147.07 |
| var(_cons[id]) | 8.41e+35 | 1.67e+32 |
|  | 1.21e+37 | 2.30e+33 |
| cov(t[id],_cons[id]) | 0.00 | 0.00 |
|  | 0.00 | 0.00 |
| Statistics |  |  |
| N | 6240 | 6240 |
| ll | -2394.30 | -2165.64 |
| chi2 | 218.50 | 241.77 |
| aic | 4874.61 | 4417.29 |

Models for both outcomes were estimated with mixed-effects logit with patient-level random effects and unstructured covariance. Reported ẞ’s are odds ratios. Restricted to a maximum FU is 5 years.

Key: * p<0.05; ** p<0.01; *** p<0.001

## Supplementary Table 9. Associations between missingness and outcomes

| Outcome | Any missings in QALYs (ẞ/se) | | | | | Any missings in costs (ẞ/se) | | | |
| --- | --- | --- | --- | --- | --- | --- | --- | --- | --- |
| FU year | 1 | 2 | 3 | 4 | 5 | 2 | 3 | 4 | 5 |
| Utility at baseline | 0.81 | 0.94 | 0.66 | 0.57 | 4.69 | 0.53 | 0.39 | 0.48 | 4.64 |
|  | 0.49 | 0.70 | 0.43 | 0.48 | 9.76 | 0.65 | 0.27 | 0.40 | 8.49 |
| Costs FU year 1 |  | 1.00 | 1.00* | 1.00 | 1.00* | 1.00 | 1.00** | 1.00 | 1.00 |
|  |  | 0.00 | 0.00 | 0.00 | 0.00 | 0.00 | 0.00 | 0.00 | 0.00 |
| QALYs FU year 1 |  | 0.44 | 0.65 | 0.50 | 106.57 | 0.13 | 1.52 | 0.42 | 15.73 |
|  |  | 0.38 | 0.67 | 0.64 | 317.98 | 0.19 | 1.76 | 0.53 | 42.70 |
| Costs FU year 2 |  |  | 1.00 | 1.00 | 1.00 |  | 1.00 | 1.00 | 1.00 |
|  |  |  | 0.00 | 0.00 | 0.00 |  | 0.00 | 0.00 | 0.00 |
| QALYs FU year 2 |  |  | 1.23 | 3.91 | 0.00 |  | 1.58 | 7.93 | 0.00 |
|  |  |  | 1.11 | 5.03 | 0.00* |  | 1.58 | 10.24 | 0.01 |
| Costs FU year 3 |  |  |  | 1.00 | 1.00 |  |  | 1.00 | 1.00 |
|  |  |  |  | 0.00 | 0.00 |  |  | 0.00 | 0.00 |
| QALYs FU year 3 |  |  |  | 1.03 | 16.78 |  |  | 0.72 | 28.21 |
|  |  |  |  | 1.15 | 50.13 |  |  | 0.79 | 76.40 |
| Costs FU year 4 |  |  |  |  | 1.00 |  |  |  | 1.00 |
|  |  |  |  |  | 0.00 |  |  |  | 0.00 |
| QALYs FU year |  |  |  |  | 0.30 |  |  |  | 0.20 |
|  |  |  |  |  | 0.77 |  |  |  | 0.47 |
| N | 1248 | 1074 | 904 | 604 | 240 | 1074 | 904 | 604 | 240 |
| ll | -475.96 | -459.05 | -562.05 | -405.81 | -98.89 | -161.63 | -501.30 | -413.40 | -116.13 |
| chi2 | 0.12 | 3.29 | 7.34 | 4.06 | 15.24 | 5.34 | 9.67 | 6.70 | 13.37 |
| aic | 955.91 | 926.10 | 1136.10 | 827.62 | 217.78 | 331.26 | 1014.61 | 842.80 | 252.26 |

Models for both outcomes were estimated with mixed-effects logit with patient-level random effects and unstructured covariance. Reported ẞ’s are odds ratios. Restricted to a maximum FU is 5 years.

Key: * p<0.05; ** p<0.01; *** p<0.001

## Supplementary Table 10. Testing sample heterogeneity: total QALYs, and total costs at average FU by country and arm

| Outcome | Arm | Total QALYs (n) | | Total costs (EUR) | |
| --- | --- | --- | --- | --- | --- |
| Country/ metric |  | N | Mean | N | Mean |
| Germany | PAL+ET | 197 | 2.540 | 205 | 42304 |
| Germany | ET | 190 | 2.231 | 202 | 9381 |
| Spain | PAL+ET | 106 | 2.137 | 120 | 42038 |
| Spain | ET | 126 | 2.093 | 129 | 6175 |
| France | PAL+ET | 50 | 1.912 | 56 | 39516 |
| France | ET | 57 | 1.891 | 62 | 5196 |
| Austria | PAL+ET | 16 | 2.465 | 17 | 40653 |
| Austria | ET | 8 | 2.113 | 8 | 14056 |
| Ireland, UK | PAL+ET | 14 | 2.338 | 18 | 32524 |
| Ireland, UK | ET | 29 | 2.240 | 29 | 4750 |
| USA | PAL+ET | 78 | 2.264 | 81 | 40256 |
| USA | ET | 75 | 2.187 | 75 | 6616 |
| Australia | PAL+ET | 46 | 2.534 | 47 | 45196 |
| Australia | ET | 40 | 2.121 | 42 | 7179 |
| Japan | PAL+ET | 25 | 3.223 | 25 | 41818 |
| Japan | ET | 20 | 2.803 | 20 | 9592 |
| S. Korea | PAL+ET | 25 | 3.021 | 25 | 44239 |
| S. Korea | ET | 21 | 2.837 | 21 | 4902 |
| H0: outcomes are homogeneous between countries | | | | | |
| Q statistic |  |  | NA |  | NA |
| H statistic |  |  | 4.81 |  | 7.20 |

Critical value for Q-statistic for the likelihood test at 5% significance for 9 groups is 10.15 (Table 1 in Gail and Simon [13]). Critical value for H statistic for the upper-tail 0.95 based on Chi squared distribution with 8 degrees of freedom is 15.51. For the qualitative test (differences in the direction of the effect), H0 is rejected if Q is ≤ critical value, for the quantitative test (differences in the magnitude of the effect) H0 is rejected if H ≥ critical value. See Gail and Simon [13] for further details. Q-statistic set to NA since the effect of PAL is positive and costs are strictly higher in all countries.

## Supplementary Table 11. Model selection: QALYs

| Model | Model 1 | Model 2 | Model 3 | Model 4 | Model 5 | Model 6 | Model 7 |
| --- | --- | --- | --- | --- | --- | --- | --- |
| Covariates | ẞ/se | ẞ/se | ẞ/se | ẞ/se | ẞ/se | ẞ/se | ẞ/se |
| PAL+ET | 0.01 | 0.01 | 0.01 | 0.00 | 0.00 | 0.00 | 0.00 |
|  | 0.22 | 0.30 | 0.29 | 0.96 | 0.96 | 0.94 | 0.87 |
| FU year 2 | -0.05*** | -0.05*** | -0.05*** | -0.05*** | -0.05*** | -0.05*** | -0.05*** |
|  | 0.00 | 0.00 | 0.00 | 0.00 | 0.00 | 0.00 | 0.00 |
| FU year 3 | -0.11*** | -0.11*** | -0.11*** | -0.11*** | -0.11*** | -0.11*** | -0.11*** |
|  | 0.00 | 0.00 | 0.00 | 0.00 | 0.00 | 0.00 | 0.00 |
| FU year 4 | -0.17*** | -0.17*** | -0.17*** | -0.18*** | -0.18*** | -0.18*** | -0.18*** |
|  | 0.00 | 0.00 | 0.00 | 0.00 | 0.00 | 0.00 | 0.00 |
| FU year 5 | -0.25*** | -0.25*** | -0.25*** | -0.26*** | -0.26*** | -0.26*** | -0.26*** |
|  | 0.00 | 0.00 | 0.00 | 0.00 | 0.00 | 0.00 | 0.00 |
| PAL+ET# FU year 2 | 0.01 | 0.01 | 0.01 | 0.01 | 0.01 | 0.01 | 0.01 |
|  | 0.19 | 0.18 | 0.19 | 0.12 | 0.12 | 0.12 | 0.12 |
| PAL+ET# FU year 3 | 0.04*** | 0.04*** | 0.04*** | 0.04*** | 0.04*** | 0.04*** | 0.04*** |
|  | 0.01 | 0.01 | 0.01 | 0.00 | 0.00 | 0.00 | 0.00 |
| PAL+ET# FU year 4 | 0.05** | 0.05** | 0.05** | 0.05*** | 0.05*** | 0.05*** | 0.05*** |
|  | 0.01 | 0.01 | 0.01 | 0.01 | 0.01 | 0.01 | 0.01 |
| PAL+ET# FU year 5 | 0.09*** | 0.09*** | 0.10*** | 0.10*** | 0.10*** | 0.10*** | 0.10*** |
|  | 0.00 | 0.00 | 0.00 | 0.00 | 0.00 | 0.00 | 0.00 |
| Spain |  | -0.02** | -0.02** | -0.02** | -0.02** | -0.02** | -0.02 |
|  |  | 0.04 | 0.04 | 0.01 | 0.01 | 0.02 | 0.03 |
| France |  | -0.01 | -0.01 | 0.00 | 0.00 | 0.00 | 0.00 |
|  |  | 0.44 | 0.32 | 0.84 | 0.84 | 0.98 | 0.96 |
| Austria |  | 0.01 | 0.01 | 0.01 | 0.01 | 0.01 | 0.02 |
|  |  | 0.68 | 0.82 | 0.55 | 0.55 | 0.53 | 0.47 |
| Ireland, UK |  | 0.03 | 0.03 | 0.02 | 0.02 | 0.02 | 0.03* |
|  |  | 0.19 | 0.15 | 0.20 | 0.20 | 0.16 | 0.06 |
| USA |  | 0.02* | 0.02* | 0.03* | 0.03*** | 0.04*** | 0.05*** |
|  |  | 0.06 | 0.06 | 0.00 | 0.00 | 0.00 | 0.00 |
| Australia |  | 0.02 | 0.02 | 0.01 | 0.01 | 0.01 | 0.02* |
|  |  | 0.21 | 0.27 | 0.38 | 0.38 | 0.27 | 0.10 |
| Japan |  | 0.06*** | 0.05** | 0.04** | 0.04** | 0.04** | 0.05*** |
|  |  | 0.01 | 0.02 | 0.02 | 0.02 | 0.02 | 0.00 |
| South Korea |  | 0.03 | 0.02 | 0.02 | 0.02 | 0.02 | 0.02 |
|  |  | 0.21 | 0.31 | 0.14 | 0.14 | 0.16 | 0.17 |
| Lymph node status after surgery (ypN 0-1 vs ypN2-3) |  |  | 0.02*** | 0.02*** | 0.02** | 0.02** | 0.01** |
|  |  |  | 0.01 | 0.01 | 0.01 | 0.01 | 0.02 |
| Age at first diagnosis ≤50 |  |  | -0.01 | 0.00 | 0.00 | -0.01 | -0.02** |
|  |  |  | 0.54 | 0.61 | 0.61 | 0.13 | 0.02 |
| Ki-67 >15% |  |  | -0.03*** | -0.02*** | -0.02*** | -0.02** | -0.02*** |
|  |  |  | 0.00 | 0.00 | 0.00 | 0.00 | 0.00 |
| CPS-EG score >=3 |  |  | 0.00 | 0.00 | 0.00 | 0.00 | 0.00 |
|  |  |  | 0.81 | 0.65 | 0.68 | 0.52 | 0.58 |
| Baseline utlity |  |  |  | 0.51*** | 0.51*** | 0.51*** | 0.49*** |
|  |  |  |  | 0.00 | 0.00 | 0.00 | 0.00 |
| Tumor grade 1 or 2 |  |  |  |  | 0.00 |  |  |
|  |  |  |  |  | 0.95 |  |  |
| First line ET with Tamoxifen |  |  |  |  |  | 0.01 | 0.01 |
|  |  |  |  |  |  | 0.11 | 0.20 |
| Goserelin injections |  |  |  |  |  | 0.00 | 0.00 |
|  |  |  |  |  |  | 0.62 | 0.80 |
| Had a mastectomy |  |  |  |  |  | 0.00 | 0.00 |
|  |  |  |  |  |  | 0.50 | 0.58 |
| Had a breast reconstruction surgery |  |  |  |  |  | 0.01 | 0.01 |
|  |  |  |  |  |  | 0.32 | 0.28 |
| Number of on-going illnesses w treatment |  |  |  |  |  |  |  |
| 1 |  |  |  |  |  |  | -0.02** |
|  |  |  |  |  |  |  | 0.05 |
| 2 |  |  |  |  |  |  | -0.03*** |
|  |  |  |  |  |  |  | 0.00 |
| 3 |  |  |  |  |  |  | -0.05*** |
|  |  |  |  |  |  |  | 0.00 |
| N | 3057 | 3057 | 3057 | 3057 | 3057 | 3057 | 3057 |
| ll | 2019.17 | 2006.29 | 2001.22 | 2183.28 | 2179.34 | 2169.85 | 2171.56 |
| chi2 | 330.27 | 356.59 | 380.36 | 875.83 | 875.12 | 879.30 | 922.27 |
| aic | -3988.33 | -3946.58 | -3928.45 | -4290.57 | -4280.68 | -4255.71 | -4253.12 |

ẞ/se obtained by fitting MMRM model with unstructured covariance to trial data restricted to 5 years of FU. The preferred specification (with lowest AIC score) is highlighted in light grey.

Key: FU, follow-up; MMRM; mixed model for repeated measures; QALYs, quality-adjusted life-years.

## Supplementary Table 12. Model selection: costs

| Model | Model 1 | Model 2 | Model 3 | Model 4 | Model 5 | Model 6 | Model 7 |
| --- | --- | --- | --- | --- | --- | --- | --- |
| Covariates | ẞ/se | ẞ/se | ẞ/se | ẞ/se | ẞ/se | ẞ/se | ẞ/se |
| PAL+ET | 31426*** | 31390*** | 31392*** | 31398*** | 31392*** | 31438*** | 31438*** |
|  | 402 | 402 | 401 | 401 | 401 | 400 | 400 |
| FU year 2 | -720** | -719** | -723** | -723** | -722** | -724** | -724 |
|  | 319 | 319 | 319 | 319 | 319 | 319 | 319 |
| FU year 3 | -825** | -830** | -839** | -842** | -843** | -821** | -821 |
|  | 382 | 382 | 382 | 381 | 382 | 381 | 381 |
| FU year 4 | -1323*** | -1379*** | -1356*** | -1361*** | -1360*** | -1357*** | -1362 |
|  | 409 | 410 | 410 | 410 | 410 | 408 | 408 |
| FU year 5 | -2264*** | -2406*** | -2285*** | -2293*** | -2307*** | -2230*** | -2231 |
|  | 603 | 612 | 608 | 608 | 607 | 621 | 622 |
| PAL+ET# FU year 2 | -30026*** | -30029*** | -30027*** | -30028*** | -30029*** | -30019*** | -30020 |
|  | 449 | 448 | 448 | 448 | 448 | 448 | 448 |
| PAL+ET# FU year 3 | -31371*** | -31382*** | -31380*** | -31375*** | -31377*** | -31433*** | -31435 |
|  | 534 | 534 | 533 | 533 | 533 | 533 | 533 |
| PAL+ET# FU year 4 | -30925*** | -30932*** | -30938*** | -30929*** | -30934*** | -30968*** | -30960 |
|  | 572 | 573 | 573 | 573 | 573 | 570 | 571 |
| PAL+ET# FU year 5 | -30891*** | -30908*** | -31042*** | -31027*** | -31051*** | -31046*** | -31027 |
|  | 845 | 851 | 839 | 840 | 838 | 858 | 860 |
| Spain |  | -308 | -291 | -313 | -317 | -515* | -522* |
|  |  | 287 | 284 | 284 | 284 | 283 | 283 |
| France |  | -869** | -877** | -898** | -887** | -785** | -786** |
|  |  | 396 | 393 | 393 | 393 | 388 | 389 |
| Austria |  | -84 | 189 | 177 | 208 | -238 | -247 |
|  |  | 741 | 734 | 734 | 735 | 726 | 728 |
| Ireland, UK |  | -1506*** | -1629*** | -1631*** | -1655*** | -1557*** | -1561*** |
|  |  | 624 | 619 | 619 | 619 | 606 | 608 |
| USA |  | -500 | -509 | -538 | -537 | -854** | -822** |
|  |  | 361 | 359 | 359 | 359 | 362 | 376 |
| Australia |  | 335 | 277 | 289 | 334 | -69 | -72 |
|  |  | 434 | 430 | 430 | 433 | 431 | 435 |
| Japan |  | -183 | -65 | -52 | -80 | -546 | -551 |
|  |  | 554 | 549 | 549 | 549 | 549 | 553 |
| South Korea |  | -484 | -760 | -780 | -794 | -613 | -593 |
|  |  | 536 | 533 | 534 | 534 | 527 | 528 |
| Lymph node status after surgery (ypN 0-1 vs ypN2-3) |  |  | -389* | -389* | -360* | -170 | -170 |
|  |  |  | 210 | 210 | 213 | 208 | 209 |
| Age at first diagnosis ≤50 |  |  | 1134*** | 1138*** | 1144** | 297 | 292 |
|  |  |  | 214 | 215 | 215 | 289 | 294 |
| Ki-67 >15% |  |  | 49 | 37 | 69 | 103 | 93 |
|  |  |  | 232 | 232 | 234 | 228 | 229 |
| CPS-EG score >=3 |  |  | -206 | -211 | -86 | -167 | -171 |
|  |  |  | 222 | 222 | 259 | 218 | 218 |
| Baseline utility |  |  |  | -1028 | -1021 | -1050 | -1004 |
|  |  |  |  | 789 | 789 | 777 | 790 |
| Tumor grade 1 or 2 |  |  |  |  | 235 |  |  |
|  |  |  |  |  | 251 |  |  |
| First line ET with Tamoxifen |  |  |  |  |  | 338 | 352 |
|  |  |  |  |  |  | 274 | 276 |
| Goserelin injections |  |  |  |  |  | 2118*** | 2138*** |
|  |  |  |  |  |  | 317 | 318 |
| Had a mastectomy |  |  |  |  |  | 584*** | 580*** |
|  |  |  |  |  |  | 224 | 225 |
| Had a breast reconstruction surgery |  |  |  |  |  | 220 | 222 |
|  |  |  |  |  |  | 287 | 288 |
| Number of on-going illnesses w treatment |  |  |  |  |  |  |  |
| 1 |  |  |  |  |  |  | 132 |
|  |  |  |  |  |  |  | 258 |
| 2 |  |  |  |  |  |  | 224 |
|  |  |  |  |  |  |  | 322 |
| 3 |  |  |  |  |  |  | -124 |
|  |  |  |  |  |  |  | 358 |
| N | 3680 | 3680 | 3680 | 3680 | 3680 | 3680 | 3680 |
| ll | -36761 | -36698 | -36658 | -36649 | -36642 | -36596 | -36576 |
| chi2 | 12200 | 12230 | 12292 | 12303 | 12309 | 12371 | 12369 |
| aic | 73572 | 73463 | 73389 | 73374 | 73363 | 73277 | 73242 |

ẞ/se obtained by fitting MMRM model with unstructured covariance to trial data restricted to 5 years of FU. The preferred specification (with lowest AIC score) is highlighted in light grey.

Key: FU, follow-up; MMRM; mixed model for repeated measures; QALYs, quality-adjusted life-years.

# Supplementary File 2. Technical details on MICE implementation and diagnostics

Assuming the missings were MAR, we employed Multiple Imputation by Chained Equations (MICE) with predictive mean matching (PMM) to impute missing values in costs and QALYs by study arm. PMM was preferred to deal with skewness in the distribution of outcomes [14]. With the exception of PAL, missings in costs for all other care episodes followed the same pattern (Supplementary Table 6). Therefore, missing values were imputed at the level of interval total (i.e. sum of all care episodes in a given interval) rather than separately by type of care episode. Outcomes were imputed by year. The imputation model inculded yearly outcomes, covariates used in the main analysis (i.e. risk stratification factors, baseline health utility, and country; cost models additionally controlled for breast cancer treatments received before randomization (first ET with tamoxifen, ovarian suppression with goserelin injections, mastectomy, and reconstruction surgery) and the number of health conditions with on-going treatment (0, 1, 2, 3 and more)). For both outcomes, the MI model also included indicators for several high-cost and high burden chronic conditions such as cardiac disorders, vascular disorders, and psychiatric disorders; included baseline covariates, yearly costs and QALYs for each of the 4 years of FU. Reflecting the fraction of missings in the sample (at FU year 4 total missings were 28% for costs and 38% for QALYs (Supplementary Table 6)) MI was run with 40 imputations. We set the number of nearest neighbor observations to draw from at 5. Multiply imputed yearly outcomes were summed to produce totals for the 4 year trial FU.

Supplementary Figures 1-4 show that the MI models converged across outcomes and imputations, varying at random around the sample mean (for all outcomes by 100^th^ iteration, earlier for most).

Supplementary Figures 5-6 compare imputed values to the data for the first five imputations. Consistent with MAR assumption, we see some diviations between observed and imputed values, more strongly for QALYs. The differences are consistent with the missingness process. In the earllier periods missing values in outcomes are primarily due to loss to follow-up (patients that died or had an event were relatively more likely to drop out), reflecting this the imputed values are more densely consentrated in lower and middle QALYs compared to the observed data. In the later period, missings due to administrative censoring dominated (Supplementary Table 6); the complete observations are increasingly dominated by German patients that enrolled earlier in the trial and had somewhat higher risk. The MI model thus yielded imputed values that were higher than observed reflecting the relatively healthier patients censored due to later enrollment. Differences in costs are harder to observe largely due to the high variation in costs between pations; a variation that was adequately captured by the MI models.

Finally, we explored assumptions on the missingness process by systematically scaling up imputed values first only for QALYs and then both for QALYs and costs [15]. The main conclusion held in these additional runs suggesting MAR might be appropriate. See results reported as part of scenario analysis (Supplementary Table 17, scenarios 27-28).

Supplementary Figure 1. Convergence of MICE models by year: PAL+ET arm QALYs, n
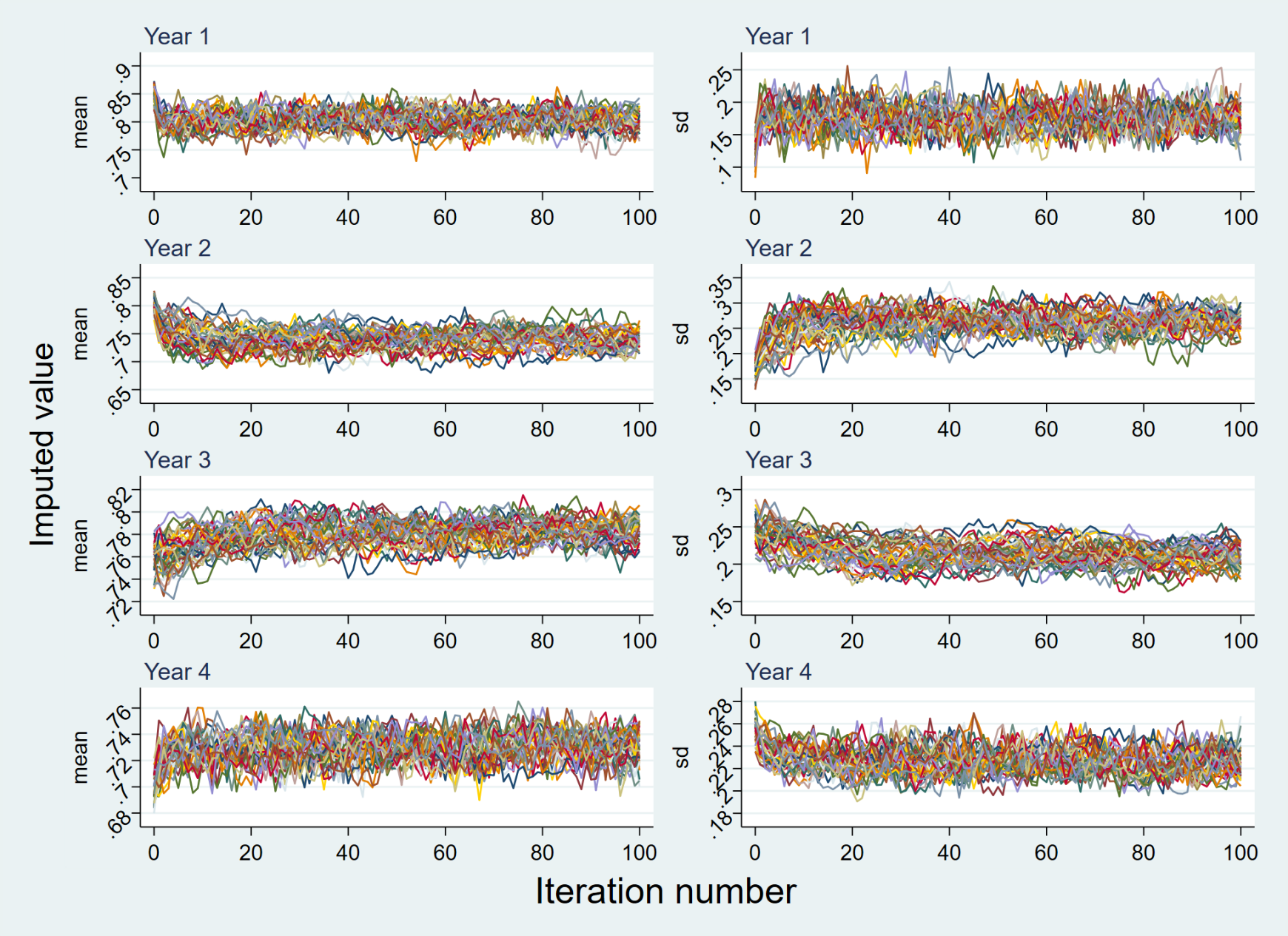


Each line represented an imputation. A total of 40 imputations matching the fraction of missing data at FU year 4 were run for the main model.

Supplementary Figure 2. Convergence of MICE models by year: ET arm QALYs, n


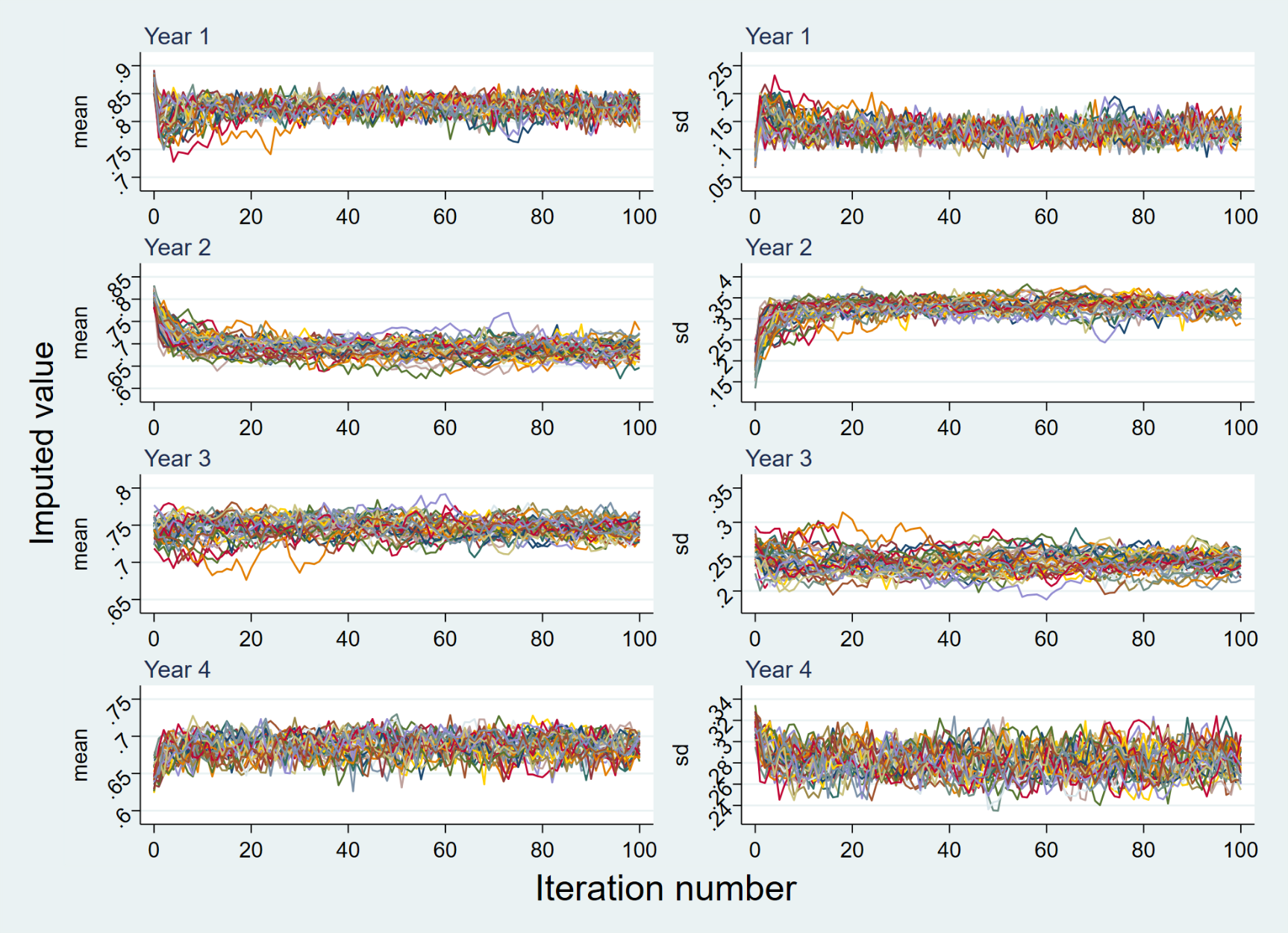


Each line represented an imputation. A total of 40 imputations matching the fraction of missing data at FU year 4 were run for the main model.

Supplementary Figure 3. Convergence of MICE models by year: PAL+ET arm costs, EUR


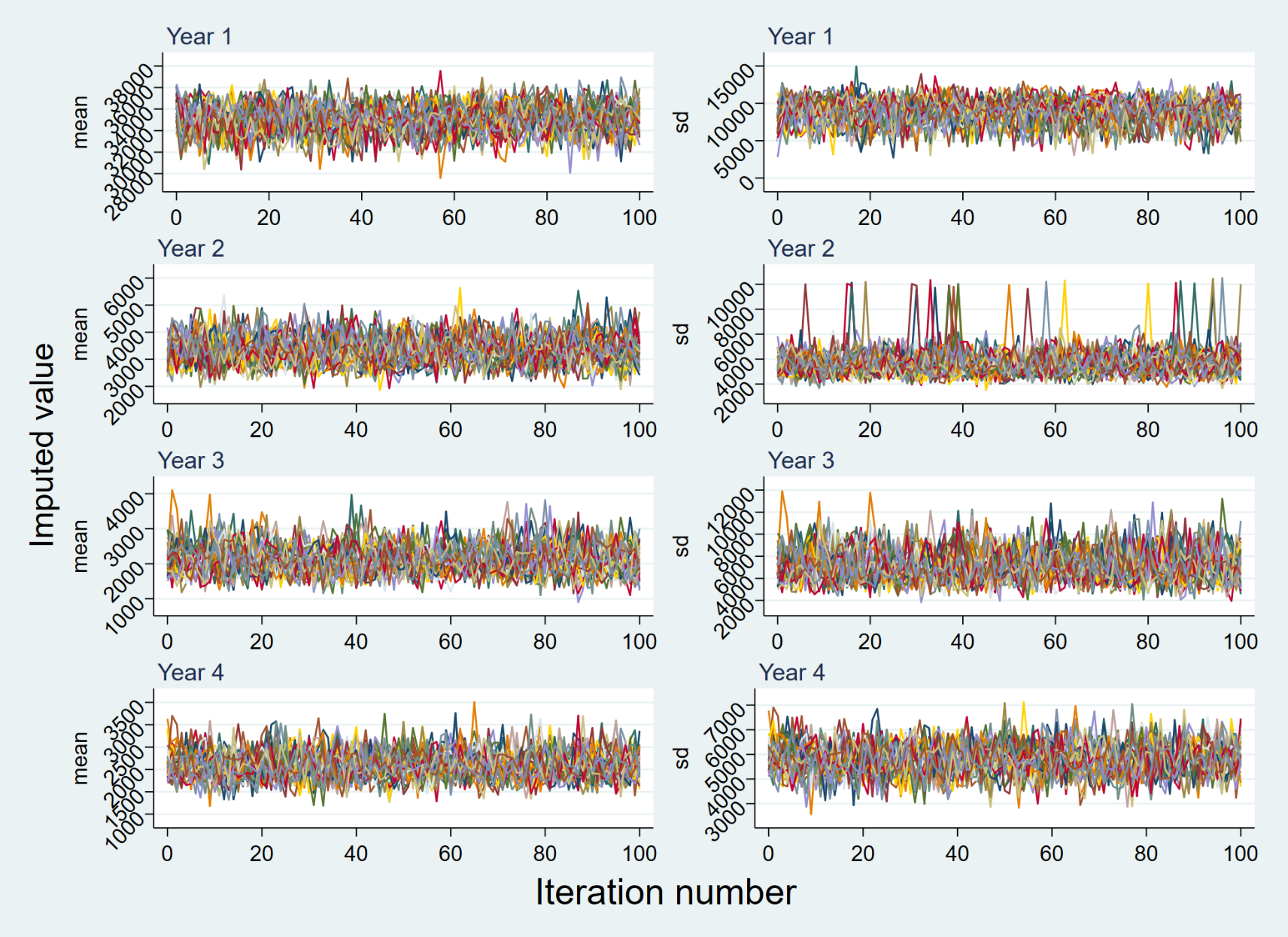


Each line represented an imputation. A total of 40 imputations matching the fraction of missing data at FU year 4 were run for the main model.

Supplementary Figure 4. Convergence of MICE models by year: ET arm costs, EUR


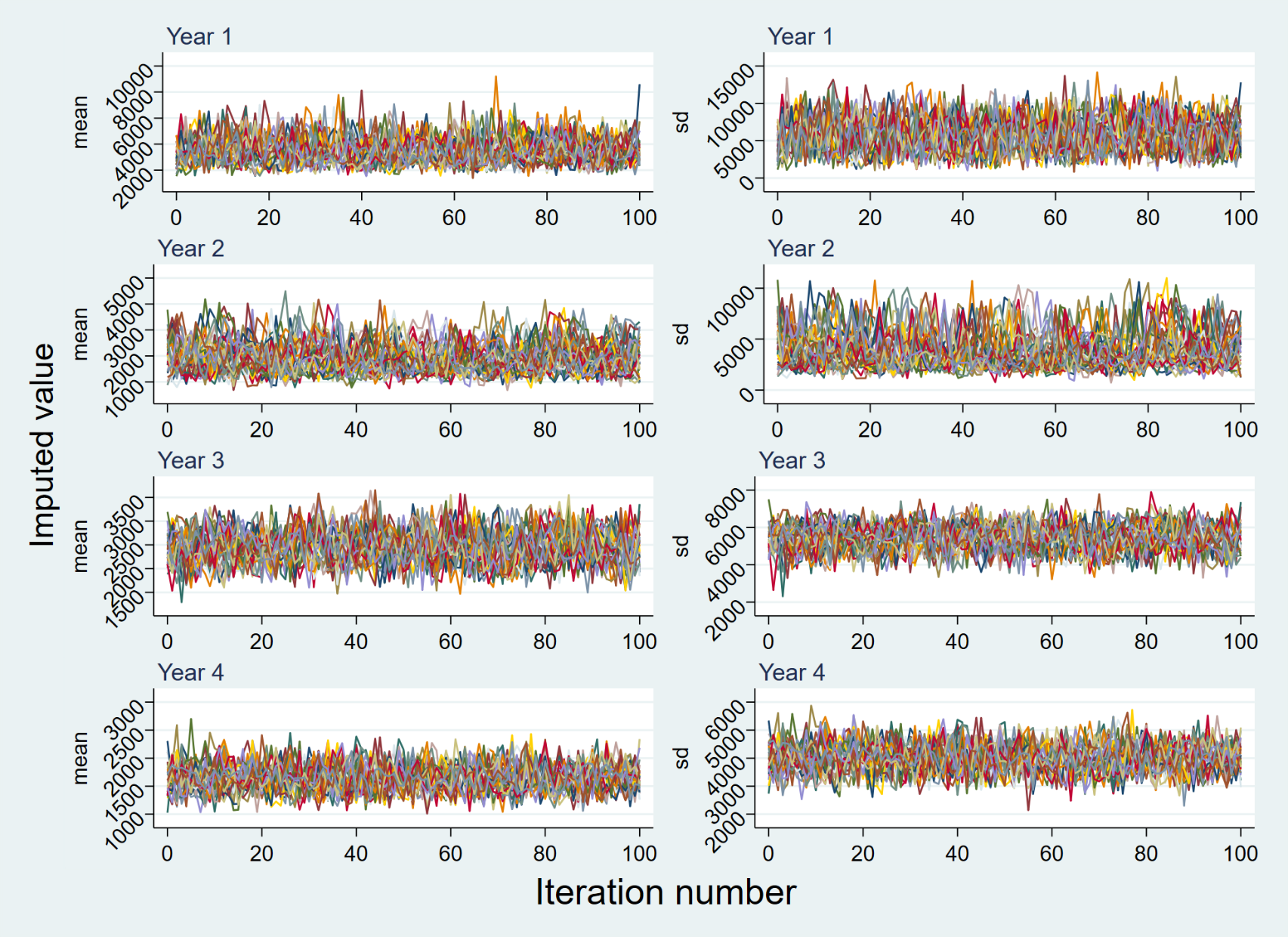


Each line represented an imputation. A total of 40 imputations matching the fraction of missing data at FU year 4 were run for the main model.

Supplementary Figure 5. Comparison of the distribution of imputed values with the observed data: QALYs imputations 1-5
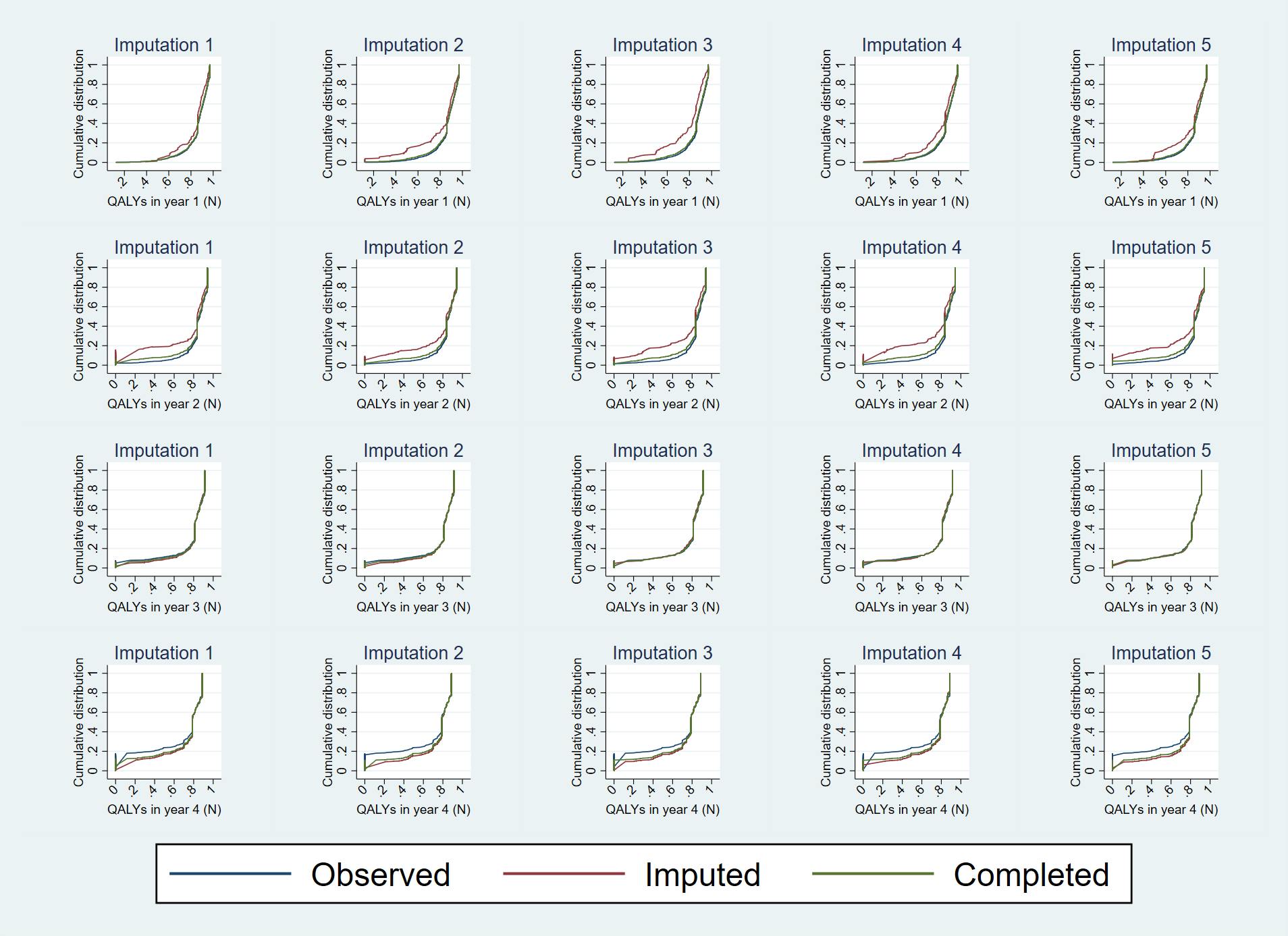


Supplementary Figure 6. Comparison of the distribution of imputed values with the observed data: costs imputations 1-5


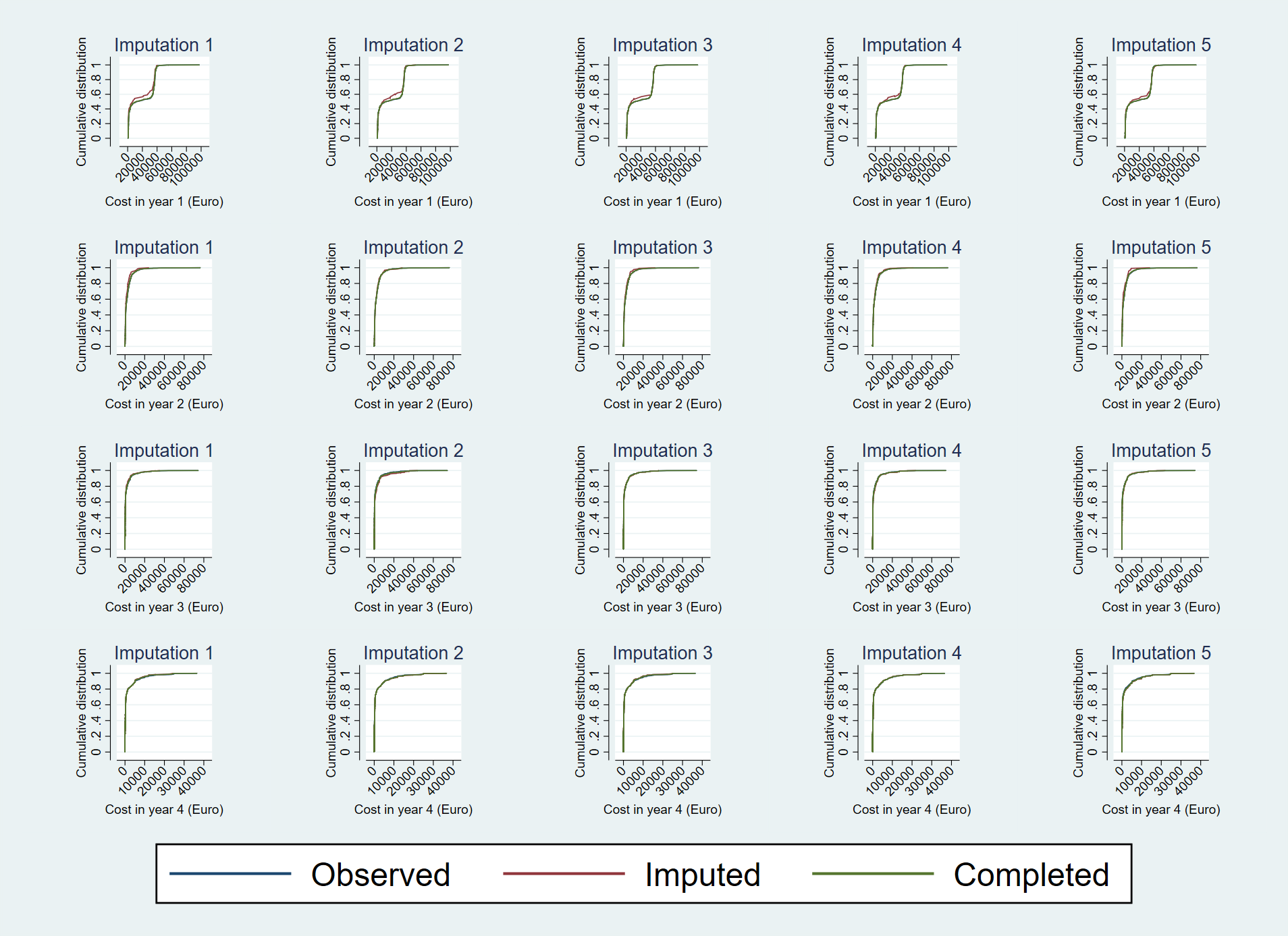


# Supplementary Material File 3. Supplementary results

## Supplementary Table 13. Patient baseline characteristics

| Population | *All countries* | | | *Germany* | | |
| --- | --- | --- | --- | --- | --- | --- |
| Arm | PAL + ET (N=631) | ET (N=619) | *P* value | PAL + ET (N=235) | ET (N=222) | P value |
| Country, % |  |  |  |  |  |  |
| Germany | 34.7 | 34.7 | 0.992 | 100 | 100 | 0 |
| Spain | 20.8 | 21.3 | 0.807 | 0 | 0 | 0 |
| USA | 13.8 | 13.6 | 0.911 | 0 | 0 | 0 |
| France | 9.4 | 10.0 | 0.691 | 0 | 0 | 0 |
| Australia | 7.6 | 6.8 | 0.574 | 0 | 0 | 0 |
| South Korea | 4.3 | 3.7 | 0.611 | 0 | 0 | 0 |
| Japan | 4.0 | 3.2 | 0.488 | 0 | 0 | 0 |
| Austria | 2.7 | 1.3 | 0.077 | 0 | 0 | 0 |
| Ireland | 2.5 | 5.0 | 0.022 | 0 | 0 | 0 |
| UK | 0.3 | 0.3 | 0.985 | 0 | 0 | 0 |
| Clinical response after NACT, % |  |  |  |  |  |  |
| CR | 8.4 | 7.9 | 0.755 | 8.7 | 7.0 | 0.510 |
| PR | 73.4 | 78.4 | 0.040 | 74.0 | 79.5 | 0.170 |
| SD | 17.3 | 12.4 | 0.016 | 16.0 | 11.6 | 0.189 |
| PD | 1.0 | 1.3 | 0.566 | 1.4 | 1.9 | 0.685 |
| Age ≤ 50, % | 56.3 | 56.5 | 0.920 | 55.7 | 57.7 | 0.679 |
| Histological nodal status at surgery ypN 0-1, % | 49.1 | 49.8 | 0.824 | 47.5 | 50.7 | 0.504 |
| CPS-EG score ≥3, % | 60.0 | 59.0 | 0.718 | 65.1 | 59.6 | 0.237 |
| Ki-67 ≤15%, % | 71.9 | 70.9 | 0.687 | 68.9 | 65.1 | 0.396 |
| Had radiotherapy, % | 98.6 | 99.0 | 0.458 | 98.6 | 99.5 | 0.324 |
| Had a mastectomy, % | 64.5 | 66.4 | 0.481 | 54.3 | 59.1 | 0.320 |
| Had a breast reconstruction surgery, % | 16.6 | 19.5 | 0.182 | 18.3 | 24.7 | 0.105 |
| Number of breast reconstruction surgeries, n | 1.23 ± 0.54 | 1.25 ± 0.55 | 0.758 | 1.35 ± 0.69 | 1.39 ± 0.67 | 0.484 |
| ET, % |  |  |  |  |  |  |
| Started ET before PAL | 89.1 | 89.7 | 0.733 | 86.3 | 86.5 | 0.949 |
| First ET with tamoxifen | 49.8 | 49.8 | 0.999 | 54.8 | 58.1 | 0.482 |
| Postmenopausal, % | 52.5 | 48.9 | 0.215 | 53.9 | 50.7 | 0.507 |
| Ovarian suppression, % |  |  |  |  |  |  |
| Goserelin injections | 14.6 | 15.3 | 0.704 | 7.8 | 12.1 | 0.131 |
| Surgical | 1.3 | 1.1 | 0.824 | 0.9 | 1.9 | 0.398 |
| Radiologic | 0.2 | 0.0 | 0.322 | 0.0 | 0.0 | <0.001 |
| Had a hysterectomy, % | 3.8 | 2.3 | 0.114 | 2.7 | 1.9 | 0.542 |
| Number of illnesses, n | 3.2 ± 3.89 | 3.44 ± 4.38 | 0.403 | 2.18 ± 2.45 | 2.19 ± 2.99 | 0.473 |
| Number of illnesses with treatment, n | 1.11 ± 1.55 | 1.12 ± 1.53 | 0.688 | 0.81 | 0.66 ± 0.98 | 0.442 |
| Had a cardiac disorder, % | 0.5 | 2.1 | 0.011 | 1.4 | 2.3 | 0.459 |
| Has a vascular disorder, % | 21.1 | 18.9 | 0.336 | 25.6 | 21.9 | 0.364 |
| Has a psychiatric disorder, % | 14.6 | 16.8 | 0.280 | 7.3 | 4.2 | 0.163 |

Continuous variables are summarized with a mean ± SD.

Key: CPS-EG, clinical pathological stage – estrogen receptor grading; CR, complete response; ECOG, Eastern Cooperative Oncology Group; ET, endocrine therapy; NACT, neoadjuvant chemotherapy; PAL, palbociclib; PD, progressive disease; PR, partial response; SD, stable disease.

## Supplementary Table 14. Distribution of average cost per patient by type of care and t, EURs

| Strategy |  | PAL+ET | | | | | | ET | | | | | |
| --- | --- | --- | --- | --- | --- | --- | --- | --- | --- | --- | --- | --- | --- |
| Type | t | 1 | 2 | 3 | 4 | 5 | 6 | 1 | 2 | 3 | 4 | 5 | 6 |
|  | N | 590 | 567 | 452 | 217 | 53 | 4 | 577 | 555 | 413 | 205 | 51 | 8 |
| PAL | mean | 32748 | 1828 | 0 | 0 | 0 | 0 | 288 | 12 | 0 | 0 | 0 | 0 |
|  | median | 35450 | 1130 | 0 | 0 | 0 | 0 | 0 | 0 | 0 | 0 | 0 | 0 |
|  | min | 0 | 0 | 0 | 0 | 0 | 0 | 0 | 0 | 0 | 0 | 0 | 0 |
|  | max | 37403 | 15824 | 0 | 0 | 0 | 0 | 37095 | 4110 | 0 | 0 | 0 | 0 |
| Hospitalizations | mean | 546 | 762 | 484 | 593 | 356 | 0 | 769 | 708 | 553 | 386 | 7 | 0 |
|  | median | 0 | 0 | 0 | 0 | 0 | 0 | 0 | 0 | 0 | 0 | 0 | 0 |
|  | min | 0 | 0 | 0 | 0 | 0 | 0 | 0 | 0 | 0 | 0 | 0 | 0 |
|  | max | 78604 | 72852 | 20130 | 14526 | 12680 | 0 | 34337 | 63266 | 33550 | 12417 | 182 | 0 |
| Screenings | mean | 128 | 87 | 81 | 95 | 87 | 0 | 137 | 81 | 73 | 57 | 45 | 0 |
|  | median | 68 | 47 | 30 | 30 | 16 | 0 | 68 | 47 | 30 | 16 | 0 | 0 |
|  | min | 0 | 0 | 0 | 0 | 0 | 0 | 0 | 0 | 0 | 0 | 0 | 0 |
|  | max | 1835 | 1469 | 1609 | 1372 | 1690 | 0 | 1268 | 692 | 1090 | 1009 | 392 | 0 |
| Physician visits | mean | 1311 | 310 | 203 | 210 | 162 | 0 | 906 | 288 | 203 | 160 | 121 | 6 |
|  | median | 1180 | 302 | 201 | 201 | 101 | 0 | 855 | 251 | 201 | 151 | 0 | 0 |
|  | min | 151 | 0 | 0 | 0 | 0 | 0 | 151 | 0 | 0 | 0 | 0 | 0 |
|  | max | 4669 | 1257 | 1106 | 955 | 754 | 0 | 2843 | 1156 | 1257 | 905 | 503 | 50 |
| Targeted therapy | mean | 121 | 183 | 680 | 759 | 430 | 0 | 307 | 578 | 696 | 527 | 332 | 0 |
|  | median | 0 | 0 | 0 | 0 | 0 | 0 | 0 | 0 | 0 | 0 | 0 | 0 |
|  | min | 0 | 0 | 0 | 0 | 0 | 0 | 0 | 0 | 0 | 0 | 0 | 0 |
|  | max | 23325 | 20962 | 46651 | 40794 | 22812 | 0 | 29080 | 40177 | 37403 | 27333 | 16955 | 0 |
| Hormone therapy | mean | 141 | 104 | 46 | 33 | 19 | 0 | 142 | 107 | 47 | 41 | 19 | 0 |
|  | median | 147 | 65 | 0 | 0 | 0 | 0 | 150 | 65 | 1 | 0 | 0 | 0 |
|  | min | 1 | 0 | 0 | 0 | 0 | 0 | 0 | 0 | 0 | 0 | 0 | 0 |
|  | max | 463 | 454 | 445 | 402 | 285 | 0 | 468 | 401 | 401 | 402 | 240 | 0 |
| Ovarian Suppression | mean | 294 | 431 | 551 | 602 | 199 | 0 | 310 | 504 | 498 | 396 | 18 | 0 |
|  | median | 0 | 0 | 0 | 0 | 0 | 0 | 0 | 0 | 0 | 0 | 0 | 0 |
|  | min | 0 | 0 | 0 | 0 | 0 | 0 | 0 | 0 | 0 | 0 | 0 | 0 |
|  | max | 3970 | 11070 | 10889 | 16333 | 5565 | 0 | 3405 | 11070 | 10889 | 10889 | 907 | 0 |
| Radiotherapy | mean | 22 | 100 | 75 | 102 | 0 | 0 | 54 | 60 | 101 | 111 | 222 | 0 |
|  | median | 0 | 0 | 0 | 0 | 0 | 0 | 0 | 0 | 0 | 0 | 0 | 0 |
|  | min | 0 | 0 | 0 | 0 | 0 | 0 | 0 | 0 | 0 | 0 | 0 | 0 |
|  | max | 9885 | 11338 | 10466 | 10757 | 0 | 0 | 6687 | 5524 | 10176 | 8140 | 6105 | 0 |
| Chemotherapy | mean | 3 | 4 | 9 | 13 | 3 | 0 | 3 | 7 | 9 | 7 | 13 | 0 |
|  | median | 0 | 0 | 0 | 0 | 0 | 0 | 0 | 0 | 0 | 0 | 0 | 0 |
|  | min | 0 | 0 | 0 | 0 | 0 | 0 | 0 | 0 | 0 | 0 | 0 | 0 |
|  | max | 279 | 385 | 725 | 450 | 164 | 0 | 459 | 432 | 518 | 364 | 267 | 0 |
| Therapy | mean | 91 | 100 | 180 | 21 | 0 | 0 | 124 | 15 | 8 | 21 | 0 | 0 |
|  | median | 0 | 0 | 0 | 0 | 0 | 0 | 0 | 0 | 0 | 0 | 0 | 0 |
|  | min | 0 | 0 | 0 | 0 | 0 | 0 | 0 | 0 | 0 | 0 | 0 | 0 |
|  | max | 11971 | 14964 | 76959 | 4466 | 0 | 0 | 12399 | 4312 | 2993 | 4312 | 0 | 0 |
| Total | mean | 590 | 567 | 452 | 217 | 53 | 4 | 577 | 555 | 413 | 205 | 51 | 8 |
|  | median | 32748 | 1828 | 0 | 0 | 0 | 0 | 288 | 12 | 0 | 0 | 0 | 0 |
|  | min | 35450 | 1130 | 0 | 0 | 0 | 0 | 0 | 0 | 0 | 0 | 0 | 0 |
|  | max | 0 | 0 | 0 | 0 | 0 | 0 | 0 | 0 | 0 | 0 | 0 | 0 |

## Supplementary Figure 7. Average annual cost per patient in the first year of FU by treatment arm, EURs


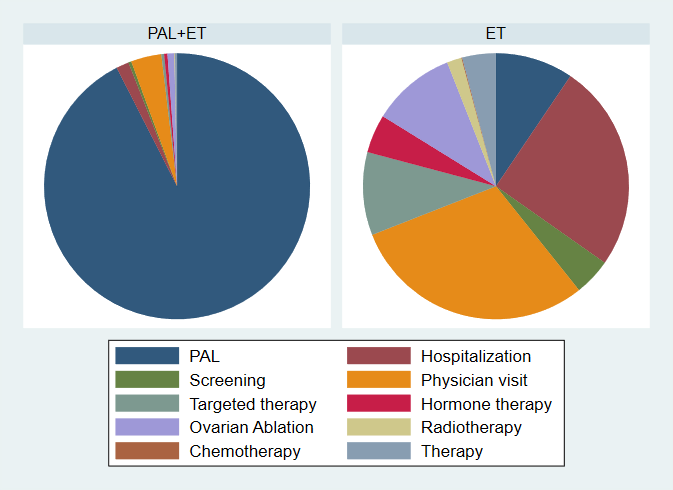


## Supplementary Figure 8. Distribution of average total cost per patient by type of care and FU year, EURs

1. *Total costs*


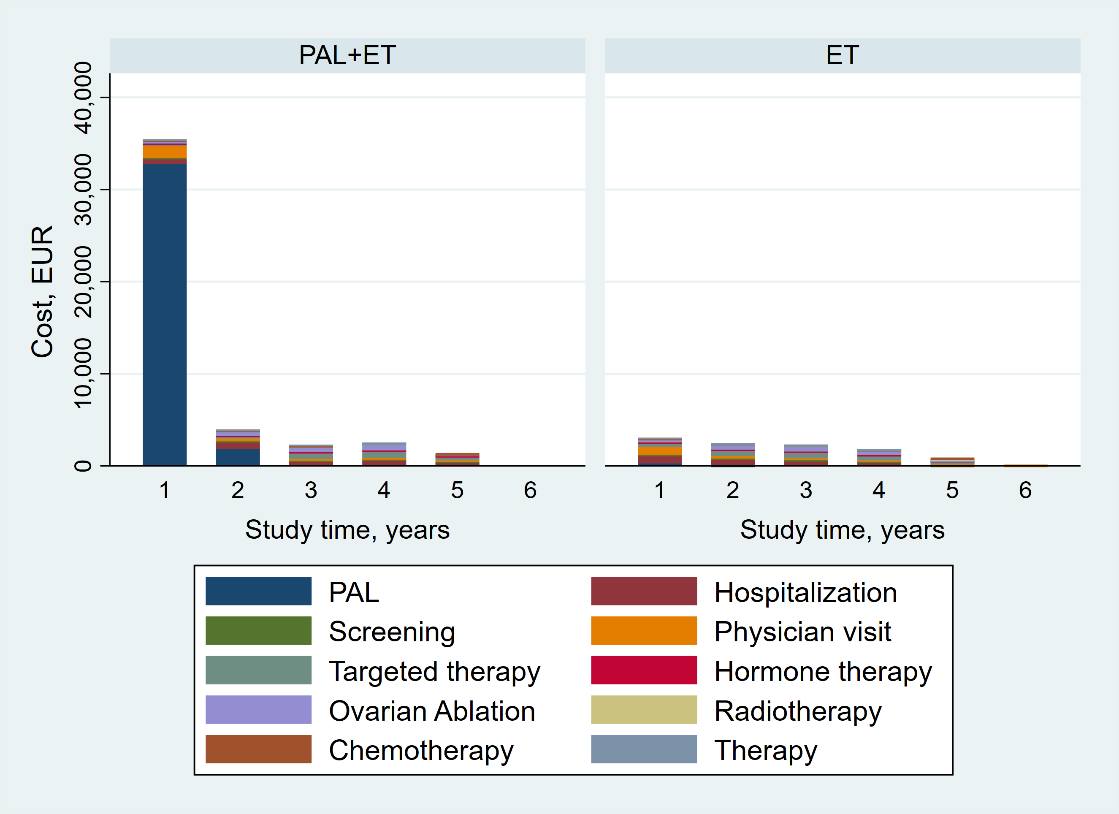


1. *Total costs net of PAL*


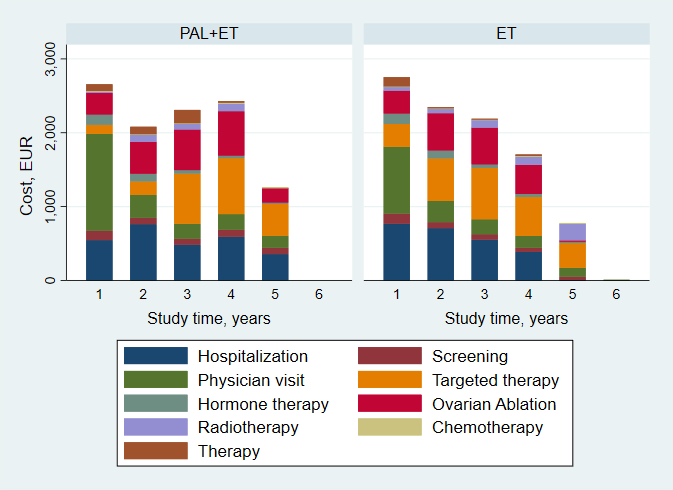


## Supplementary Figure 9. Average cost by type of care per patient and t


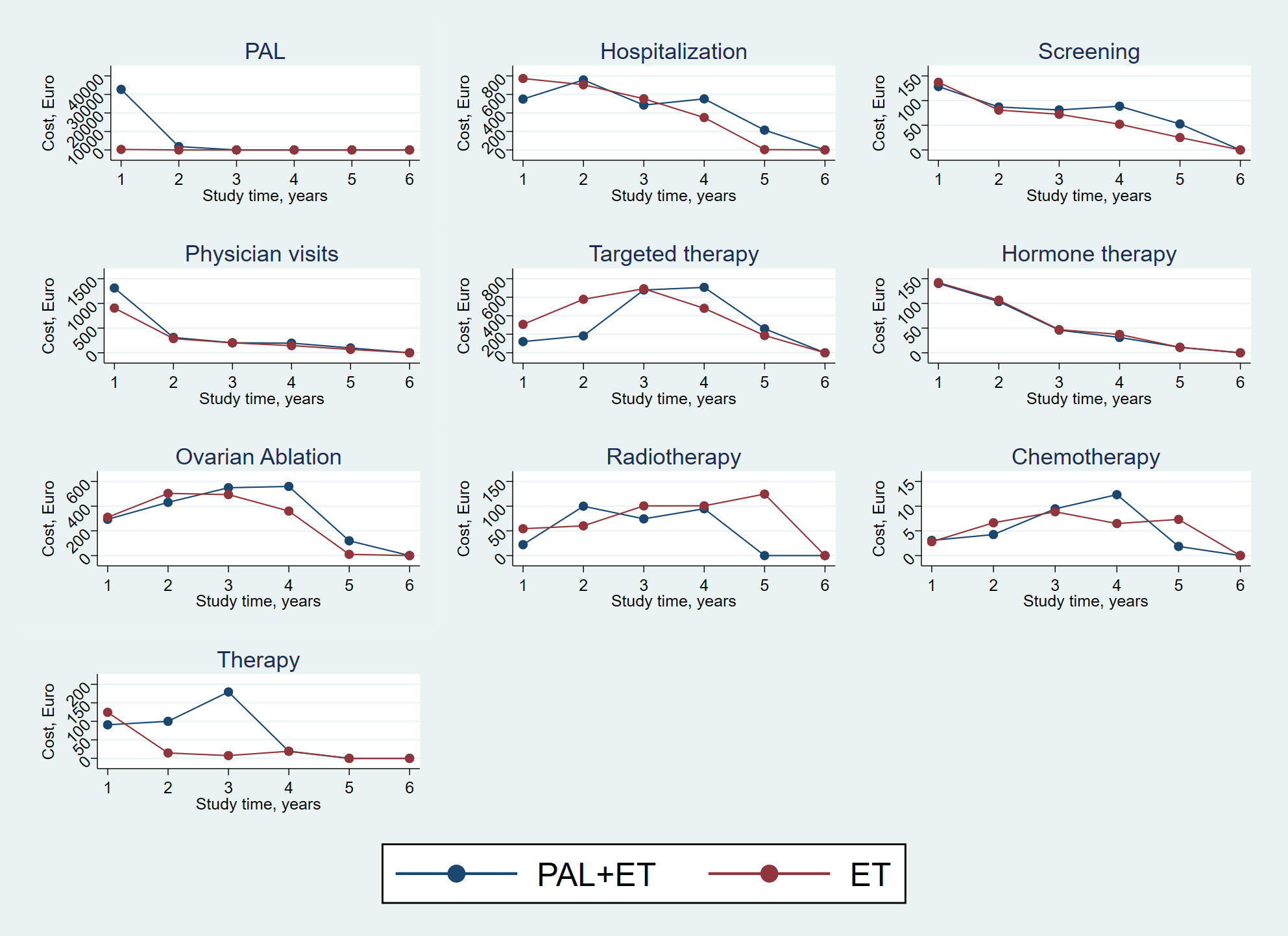


## Supplementary Figure 10. Average cost by type of care per patient and t conditional on any having any care


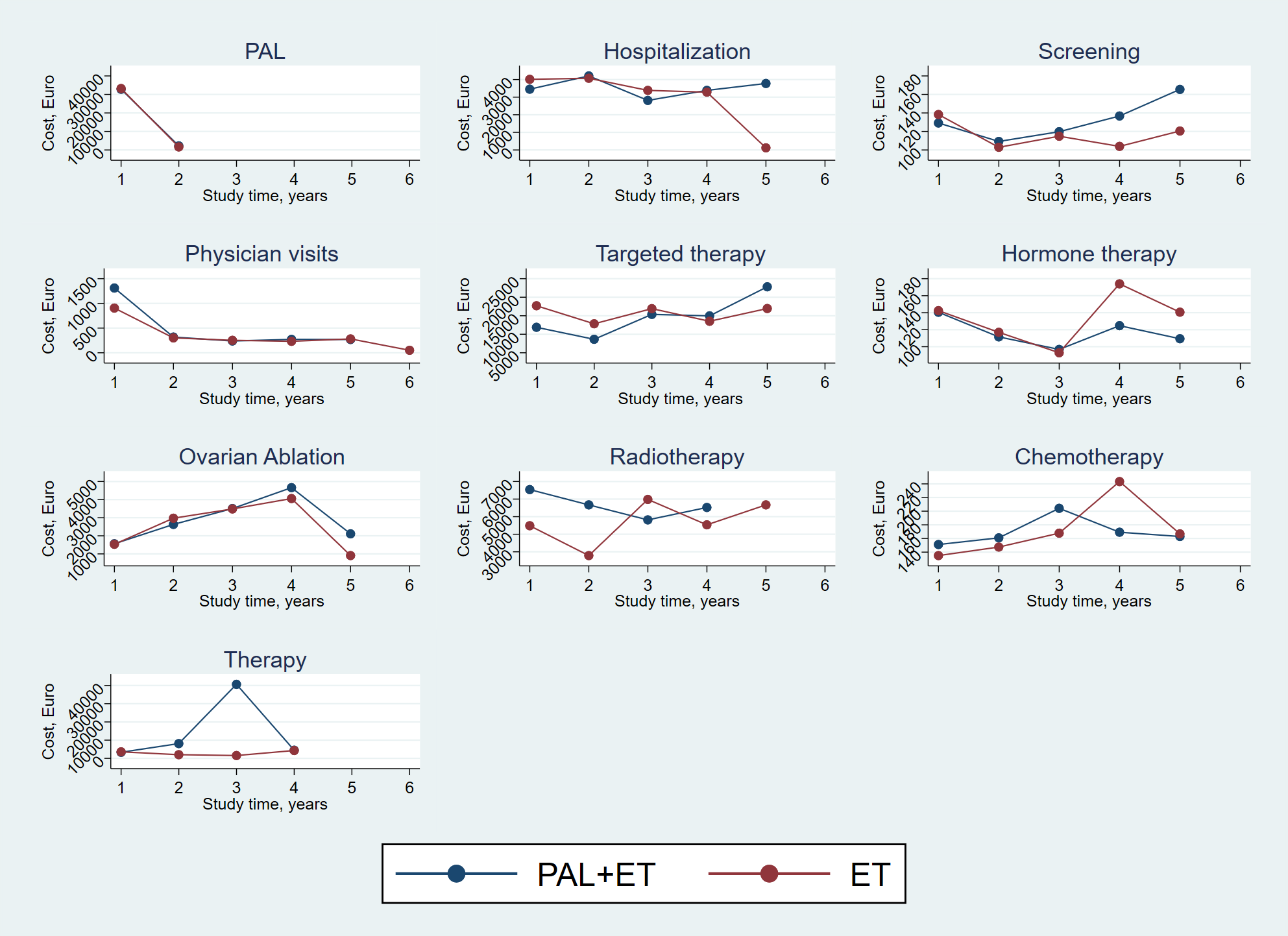


## Supplementary Figure 11. Average number of visits/ pills/ inpatient days by type of care per patient and t


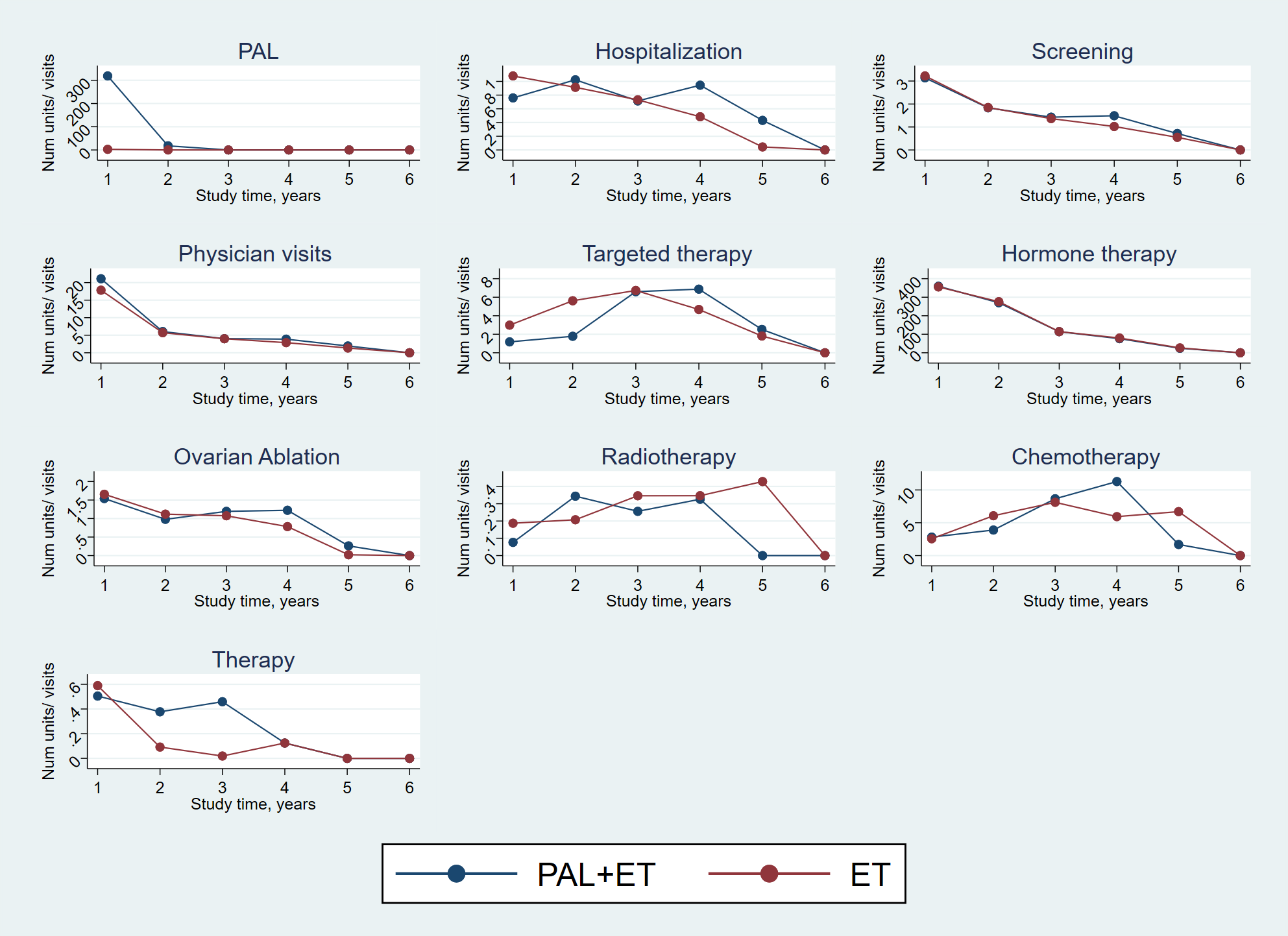


## Supplementary Figure 12. Average number of visits/ pills/ inpatient days by type of care per patient and t conditional on any having any care


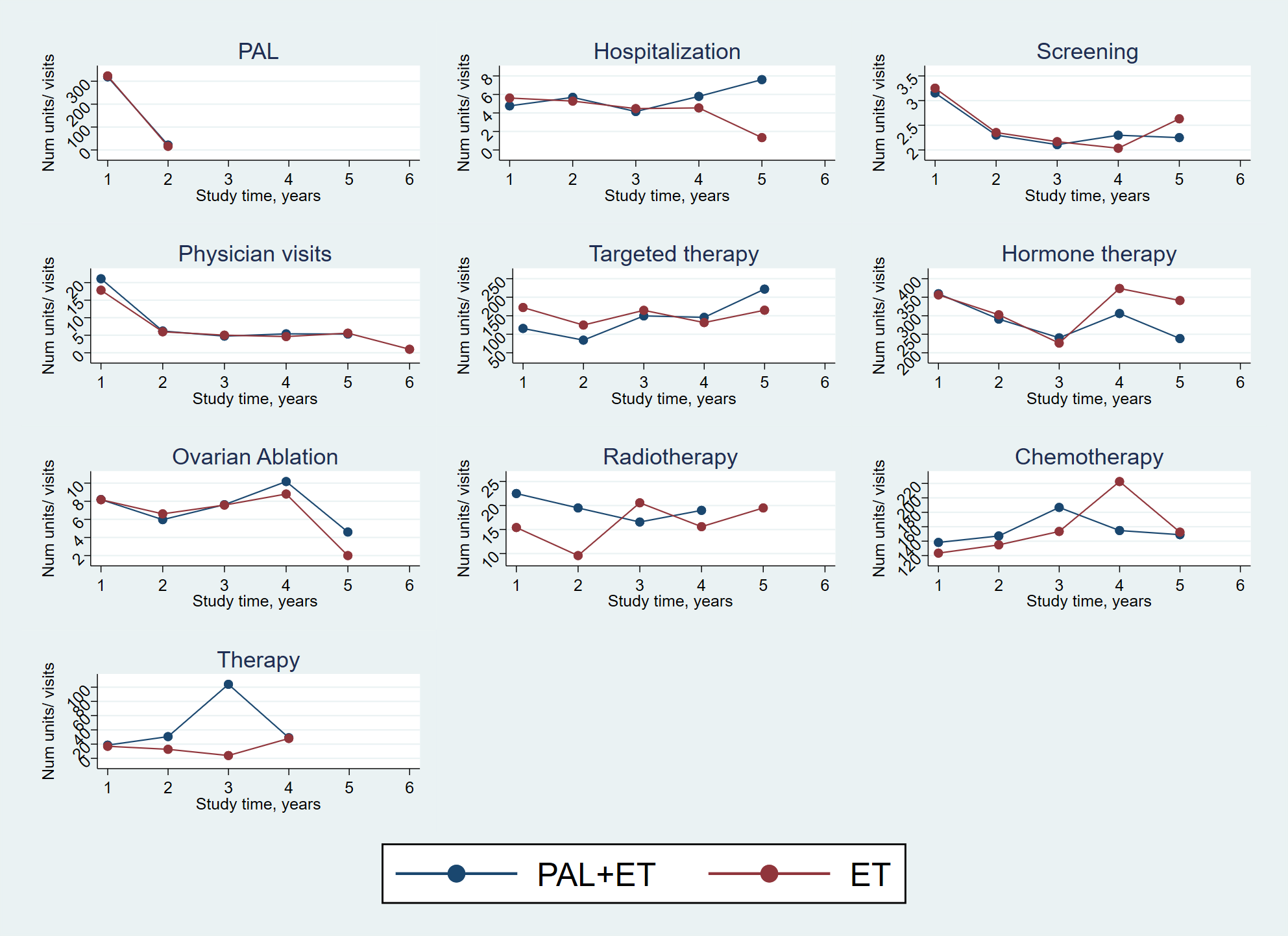


## Supplementary Table 15. Unadjusted incremental quality-adjusted life years, costs and ICER by year of FU

| FU year | Incremental QALYs, n | *P* value | Incremental costs, EUR | *P* value | ICER per QALY gained, EUR |
| --- | --- | --- | --- | --- | --- |
| 1 | 0.003 (-0.020; 0.026) | 0.795 | 31 422 (30 763; 32 081) | 0.000 | 10 474 000 |
| 2 | 0.018 (-0.007; 0.043) | 0.148 | 1 462(790; 2 134) | 0.000 | 81 222 |
| 3 | 0.042 (0.013; 0.072) | 0.005 | 111 (-655; 877) | 0.776 | 2 643 |
| 4 | 0.097 (0.052;0.141) | <0.001 | 641 (-455; 1737) | 0.252 | 6 608 |

The table presents the estimated mean (95% confidence interval) differences between the arms in QALYs gained and costs incurred in each year of FU. The data on QALYs and costs were censored to only include patients that were present or dead at the end of each yearly FU; item-missingness were relatively few and filled according to the algorithms detailed in text. The outcomes have not been adjusted to account for censoring, stratification or other imbalances.

## Supplementary Figure 13. Regression-adjusted predicted quality-adjusted life years and costs by year of FU


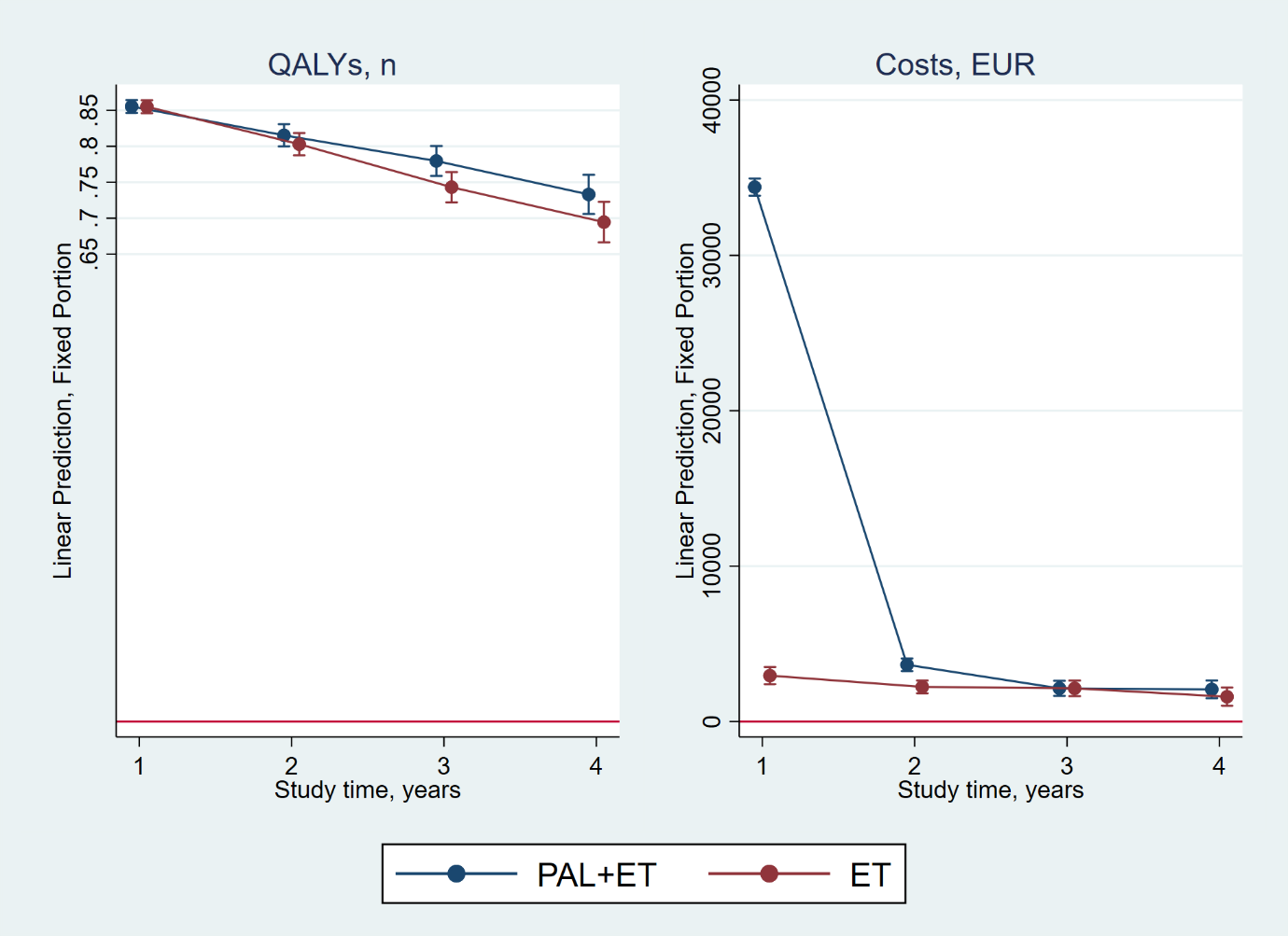


The plot presents the estimated regression-adjusted mean (95% confidence interval) differences between the arms in QALYs gained and costs incurred in each year of FU. The estimates represent average marginal effects of PAL predicted for each year of FU from mixed level linear models, see text for details. The data on QALYs and costs were censored to only include patients that were present or dead at the end of each yearly FU; item-missingness were relatively few and filled according to the algorithms detailed in text.

## Supplementary Table 16. Regression-adjusted incremental quality-adjusted life years, costs and ICER by year of FU

| FU year | Incremental QALYs, n | *P* value | Incremental costs, EUR | *P* value | ICER per QALY gained, EUR |
| --- | --- | --- | --- | --- | --- |
| 1 | 0.000 (-0.012; 0.013) | 0.959 | 31 441 (30 658; 32 224) | <0.001 | 93 371 829 |
| 2 | 0.012 (-0.009; 0.034) | 0.267 | 1 422 (840; 2 003) | <0.001 | 114 630 |
| 3 | 0.036 (0.007; 0.066) | 0.016 | 2 (-700; 704) | 0.996 | 51 |
| 4 | 0.038 (-0.001; 0.078) | 0.054 | 472 (-347; 1 291) | 0.259 | 12 253 |

The table presents the estimated regression-adjusted mean (95% confidence interval) differences between the arms in QALYs gained and costs incurred in each year of FU. The estimates represent average marginal effects of PAL predicted for each year of FU from mixed level linear models, see text for details. The data on QALYs and costs were censored to only include patients that were present or dead at the end of each yearly FU; item-missingness were relatively few and filled according to the algorithms detailed in text.

# Supplementary File 4. Scenario analyses

An extensive set of scenarios were evaluated to support the main conclusions of the paper. Supplementary Table 17 present the cumulative incremental impacts of PAL over the first four years of FU and the respective 95% confidence intervals and p-values for each scenario. We briefly present the motivation for each scenario evaluated.

We first tested sensitivity of our findings to the sample definitions aligned with clinical analyses (scenarios 1-3). We explored generalizability of the pooled sample results to Germany by restricting the analyses to patients recruited in Germany (scenario 4). We further tested performance of PAL in sub-samples stratified by risk factors (scenarios 5-12) to better appreciate the characteristics of patients that are more likely to benefit from PAL. We designed several scenarios that addressed the data limitations discussed in the methods section and in further detail in Supplementary Material 1 and relaxed some of the censoring rules we imposed to improve consistency of outcomes summarized at yearly intervals (scenarios 13-23). We calculated impact estimates through year 5 to appreciate the direction of the trend; these estimates are highly uncertain due to administrative censoring (scenario 24). By year 5 the sample included 188 patients of which 73% were dead; this represents a censoring rate of 85%. We further explored sensitivity of our findings to handling of the missing data comparing our mixed-effects model results to complete case analysis and model estimates based on multiply imputed data (scenarios 25-26). We tested MAR assumption by varying outcomes in the imputed observations by arm (scenarios 27-28). Alternative functional forms were also explored, specifically SUR model that accounts for the correlation between the two outcomes and GLM that addresses skewness in the outcomes modelled (scenarios 29-30). The two final scenarios showed the impact of discounting on the incremental impacts.

## Supplementary Table 17. Scenario analyses: regression-adjusted cumulative incremental outcomes and cost-effectiveness ratios at 4 years of FU

| No | Rationale | Scenario | Incremental QALYs, n | *P* value | Incremental costs, EUR | *P* value | ICER |
| --- | --- | --- | --- | --- | --- | --- | --- |
| 0 |  | Base case | 0.088 (-0.001; 0.177) | 0.054 | 33 336 (31 640; 35 033) | <0.001 | 378 818 |
| 1 | Target population | Per protocol population* | 0.097 (0.007; 0.186) | 0.035 | 33 381 (31 618; 35 144) | <0.001 | 344 134 |
| 2 | Target population | All randomized and treated safety population | 0.086 (-0.003; 0.174) | 0.058 | 33 904 (32 240; 35 568) | <0.001 | 394 233 |
| 3 | Target population | Evaluable patients (received 80% of PAL doses) | 0.174 (0.087; 0.261) | <0.001 | 35 974 (34 392; 37 556) | <0.001 | 206 747 |
| 4 | Between country heterogeneity | Patient recruited in Germany only | 0.021 (-0.141; 0.184) | 0.797 | 33 668 (30 308; 37 028) | <0.001 | 1 603 238 |
| 5 | Risk factor | ypN 0-1 | 0.084 (-0.035; 0.203) | 0.167 | 32 542 (30 203; 34 881) | <0.001 | 387 405 |
| 6 | Risk factor | ypN 2-3 | 0.104 (-0.029; 0.237) | 0.126 | 34 071 (31 521; 36 620) | <0.001 | 327 606 |
| 7 | Risk factor | Age ≤ 50 years | 0.034 (-0.086; 0.155) | 0.579 | 34 672 (32 267; 37 076) | <0.001 | 1 019 765 |
| 8 | Risk factor | Age > 50 years | 0.157 (0.024; 0.289) | 0.020 | 31 822 (29 468; 34 176) | <0.001 | 202 688 |
| 9 | Risk factor | Ki-67 ≤ 15% | 0.088 (-0.003; 0.179) | 0.059 | 33 873 (31 839; 35 907) | <0.001 | 384 920 |
| 10 | Risk factor | Ki-67 > 15% | 0.078 (-0.134; 0.290) | 0.472 | 31 335 (28 061; 34 610) | <0.001 | 401 731 |
| 11 | Risk factor | CPS-EG score 2 and ypN+ | 0.062 (-0.065; 0.188) | 0.341 | 33 415 (30 533; 36 297) | <0.001 | 538 952 |
| 12 | Risk factor | CPS-EG score ≥ 3 | 0.094 (-0.030; 0.219) | 0.138 | 33 401 (31 266; 35 535) | <0.001 | 355 330 |
| 13 | Outliers | Exclude patients with top 5% costs (costs net of PAL) | 0.083 (-0.008; 0.174) | 0.074 | 33 390 (32 324; 34 456) | <0.001 | 402 289 |
| 14 | Outliers | Exclude patients with bottom 5% costs (costs net of PAL) | 0.051 (-0.032; 0.134) | 0.227 | 33 280 (31 566; 34 994) | <0.001 | 652 549 |
| 15 | Data limitations | Exclude non-breast-cancer hospitalizations | 0.088 (-0.001; 0.177) | 0.054 | 33 178 (31 571; 34 786) | <0.001 | 377 023 |
| 16 | Data limitations | Include imputed expenditure in the year of death | 0.088 (-0.001; 0.177) | 0.054 | 33 293 (31 591; 34 995) | <0.001 | 378 330 |
| 17 | Data limitations | Allow for non-adherence to targeted therapy following relapse | 0.088 (-0.001; 0.177) | 0.054 | 33 649 (32 224; 35 074) | <0.001 | 382 375 |
| 18 | Data limitations | Include physician visits recorded in HE file and ignore everything else except for trial-driven assessments | 0.088 (-0.001; 0.177) | 0.054 | 32 881 (31 210; 34 551) | <0.001 | 373 648 |
| 19 | Data limitations | Exclude imputed follow-up and referral visits | 0.088 (-0.001; 0.177) | 0.054 | 33 336 (31 640; 35 033) | <0.001 | 378 818 |
| 21 | Data limitations | Keep partial year contributions (no censoring if dropped-off mid-year) | 0.006 (-0.114; 0.125) | 0.928 | 31 384 (29 828; 32 940) | <0.001 | 5 230 667 |
| 22 | Data limitations | Remove top-coding of records at study cut-off date and/ or last FU for those that never dropped out | 0.045 (-0.054; 0.144) | 0.376 | 32 623 (31 163; 34 084) | <0.001 | 724 956 |
| 23 | Data limitations | Double costs in patients that had an event in years following relapse or secondary malignancy | 0.088 (-0.001; 0.177) | 0.054 | 33 336 (31 640; 35 033) | <0.001 | 378 818 |
| 24 | Data limitations | Include data through year 5 | 0.201 (0.069; 0.332) | 0.003 | 33 749 (31 450; 36 048) | <0.001 | 167 905 |
| 25 | Missing values | Complete case analysis | -0.027 (-0.079; 0.025) | 0.311 | 34 672 (32 745; 36 598) | <0.001 | Detrimental |
| 26 | Missing values | MICE, OLS | 0.103 (0.015; 0.191) | 0.022 | 33 287 (31 655; 34 919) | <0.001 | 323 175 |
| 27 | Missing values | MICE, OLS reduce QALYs in first year by 20% and 10% in all subsequent years for imputed records | 0.097 (0.012; 0.182) | 0.025 | 33 287 (31 655; 34 919) | <0.001 | 343 165 |
| 28 | Missing values | MICE, OLS reduce QALYs as above and increase costs by the same amount | 0.097 (0.012; 0.182) | 0.025 | 33 373 (31 553; 35 193) | <0.001 | 344 052 |
| 29 | Correlation between outcomes | MICE, SUR | 0.096 (0.000; 0.192) | 0.051 | 35 070 (31 502; 38 638) | <0.001 | 365 313 |
| 30 | Skewed outcomes | MICE, GLM | 0.103 (0.015; 0.190) | 0.021 | 33 288 (31 669; 34 907) | <0.001 | 323 184 |
| 31 | Opportunity cost | 0% discount rate | 0.099 (0.001; 0.196) | 0.047 | 34 424 (32 592; 36 257) | <0.001 | 347 717 |
| 32 | Opportunity cost | 10% discount rate | 0.067 (-0.006; 0.141) | 0.073 | 31 054 (29 614; 32 493) | <0.001 | 463 493 |

The table presents the estimated regression-adjusted mean (95% confidence interval) differences between the arms in QALYs gained and costs incurred in each year of FU. The estimates represent average marginal effects of PAL predicted for each year of FU from mixed level linear models, see text for details. Unless stated otherwise, the data on QALYs and costs were censored to only include patients that were present or dead at the end of each yearly FU; item-missingness were relatively few and filled according to the algorithms detailed in text. ^*^ See Loibl et al for exclusion of patients from per protocol analysis. Scenarios 16-19 entailed multiple imputation with chained equations, missing values were filled following predictive mean matching (radius 5) to account for skewness of the data. Further details on MICE are in Supplementary Material 2.

Key: FU, follow-up; ICER, incremental cost-effectiveness Ratio; GLM, generalized linear model; OLS, ordinary least squares; PAL, palbociclib; SUR, seemingly unrelated regressions; QALYs, quality-adjusted life-years.

# Supplementary File 5. CHEERS checklist

| Section/item | Item no. | Recommendation | Reported on page |
| --- | --- | --- | --- |
| Title and abstract |  |  |  |
| Title | 1 | Identify the study as an economic evaluation, or use more specific terms such as ‘‘cost-effectiveness analysis’’ and describe the interventions compared. | 1 |
| Abstract | 2 | Provide a structured summary of objectives, perspective, setting, methods (including study design and inputs), results (including base-case and uncertainty analyses), and conclusions. | 6 |
| Introduction |  |  |  |
| Background and objectives | 3 | Provide an explicit statement of the broader context for the study. Present the study question and its relevance for health policy or practice decisions. | 7 |
| Methods |  |  |  |
| Target population and subgroups | 4 | Describe characteristics of the base-case population and subgroups analyzed including why they were chosen. | 8, 13 |
| Setting and location | 5 | State relevant aspects of the system(s) in which the decision(s) need(s) to be made. | 7-8 |
| Study perspective | 6 | Describe the perspective of the study and relate this to the costs being evaluated. | 8 |
| Comparators | 7 | Describe the interventions or strategies being compared and state why they were chosen. | 8 |
| Time horizon | 8 | State the time horizon(s) over which costs and consequences are being evaluated and say why appropriate. | 8 |
| Discount rate | 9 | Report the choice of discount rate(s) used for costs and outcomes and say why appropriate. | 8 |
| Choice of health outcomes | 10 | Describe what outcomes were used as the measure(s) of benefit in the evaluation and their relevance for the type of analysis performed. | 9 |
| Measurement of effectiveness | 11a | Single study–based estimates: Describe fully the design features of the single effectiveness study and why the single study was a sufficient source of clinical effectiveness data. | 8 |
|  | 11b | Synthesis-based estimates: Describe fully the methods used for the identification of included studies and synthesis of clinical effectiveness data. | NA |
| Measurement and valuation of preference-based outcomes | 12 | If applicable, describe the population and methods used to elicit preferences for outcomes. | 9 |
| Estimating resources and costs | 13a | Single study–based economic evaluation: Describe approaches used to estimate resource use associated with the alternative interventions. Describe primary or secondary research methods for valuing each resource item in terms of its unit cost. Describe any adjustments made to approximate to opportunity costs. | 9-10 |
|  | 13b | Model-based economic evaluation: Describe approaches and data sources used to estimate resource use associated with model health states. Describe primary or secondary research methods for valuing each resource item in terms of its unit cost. Describe any adjustments made to approximate to opportunity costs. | NA |
| Currency, price date, and | 14 | Report the dates of the estimated resource quantities and unit costs. Describe methods for adjusting estimated unit costs to the year of reported costs if necessary. Describe methods for converting costs into a common currency base and the exchange rate. | 9-10 |
| Choice of model | 15 | Describe and give reasons for the specific type of decision-analytic model used. Providing a figure to show model structure is strongly recommended. | NA |
| Assumptions | 16 | Describe all structural or other assumptions underpinning the decision-analytic model. | NA |
| Analytic methods | 17 | Describe all analytic methods supporting the evaluation. This could include methods for dealing with skewed, missing, or censored data; extrapolation methods; methods for pooling data; approaches to validate or make adjustments (e.g., half-cycle corrections) to a model; and methods for handling population heterogeneity and uncertainty. | 11-12 |
| Results |  |  |  |
| Study parameters | 18 | Report the values, ranges, references, and if used, probability distributions for all parameters. Report reasons or sources for distributions used to represent uncertainty where appropriate. Providing a table to show the input values is strongly recommended. | Supplementary Table 4 in Supplementary Material 1 |
| Incremental costs and outcomes | 19 | For each intervention, report mean values for the main categories of estimated costs and outcomes of interest, as well as mean differences between the comparator groups. If applicable, report incremental cost-effectiveness ratios. | 11-12, also see Supplementary Tables 16-17 and Supplementary Figure 7 in Supplementary File 3 |
| Characterizing uncertainty | 20a | Single study–based economic evaluation: Describe the effects of sampling uncertainty for estimated incremental cost, incremental effectiveness, and incremental cost-effectiveness, together with the impact of methodological assumptions (such as discount rate, study perspective). | 13, Figure 1 and Table 4 |
|  | 20b | Model-based economic evaluation: Describe the effects on the results of uncertainty for all input parameters, and uncertainty related to the structure of the model and assumptions. | NA |
| Characterizing heterogeneity | 21 | If applicable, report differences in costs, outcomes, or cost-effectiveness that can be explained by variations between subgroups of patients with different baseline characteristics or other observed variability in effects that are not reducible by more information. | 13, Table 4 and Supplementary Table 17 in Supplementary File 4 |
| Discussion |  |  |  |
| Discussion  Study findings, limitations, generalizability, and current knowledge | 22 | Summarize key study findings and describe how they support the conclusions reached. Discuss limitations and the generalizability of the findings and how the findings fit with current knowledge. | 16 |
| Other |  |  |  |
| Source of funding | 23 | Describe how the study was funded and the role of the funder in the identification, design, conduct, and reporting of the analysis. Describe other nonmonetary sources of support. | 2 |
| Conflict of interest | 24 | Describe any potential for conflict of interest among study contributors in accordance with journal policy. In the absence of a journal policy, we recommend authors comply with International Committee of Medical Journal Editors’ recommendations. | 2-4 |

Source: Husereau et al [16]

**References**

1. Agency for Healthcare Research and Quality. *Service line based on ICD-10-CM/PCS codes*. 2019.

2. Harbeck, N. and M. Gnant, *Breast cancer.* Lancet (London, England), 2016. **389**(10074): p. 1134-1150.

3. Waks, A.G. and E.P. Winer, *Breast Cancer Treatment: A Review.* JAMA, 2019. **321**(3): p. 288-300.

4. Pérez-Heras, Í., et al., *Post chemotherapy febrile neutropenia. Length of stay and experience in our population.* Anales de Pediatría (English Edition), 2020. **92**(3): p. 141-146.

5. Arnold, M., K. Pfeifer, and A.S. Quante, *Is risk-stratified breast cancer screening economically efficient in Germany?* Plos one, 2019. **14**(5): p. e0217213.

6. ClinicalTrials.gov, *A Study of Palbociclib in Addition to Standard Endocrine Treatment in Hormone Receptor Positive Her2 Normal Patients With Residual Disease After Neoadjuvant Chemotherapy and Surgery (PENELOPE-B)*. 2021, National Library of Medicine (US): Bethesday (MD).

7. CompuGroup Medical, *Lauer-Taxe*. 2020, CGM.

8. Kassenärztliche Bundesvereinigung, *Honorarbericht*. 2020, KBV: Berlin.

9. Federal Reserve Bank of St. Louis, *FRED Economic Data, GDP Deflator*. 2020, FRB: St Louis.

10. Deutsche Rentenversicherung, *Reha-Bericht*. 2019.

11. Institut für das Entgeltsystem im Krankenhaus, *aG-DRG-System 2020 Fallpauschalen-Katalog*. 2020, InEK: Siegburg.

12. Kassenärztliche Bundesvereinigung, *Einheitlicher Bewertungsmaßstab (EBM), Online-Version des EBM*. 2020, KBV: Berlin.

13. Gail, M. and R. Simon, *Testing for qualitative interactions between treatment effects and patient subsets.* Biometrics, 1985: p. 361-372.

14. Little, R.J., *Missing-data adjustments in large surveys.* Journal of Business & Economic Statistics, 1988. **6**(3): p. 287-296.

15. Faria, R., et al., *A guide to handling missing data in cost-effectiveness analysis conducted within randomised controlled trials.* Pharmacoeconomics, 2014. **32**(12): p. 1157-1170.

16. Husereau, D., et al., *Consolidated health economic evaluation reporting standards (CHEERS)—explanation and elaboration: a report of the ISPOR health economic evaluation publication guidelines good reporting practices task force.* Value in Health, 2013. **16**(2): p. 231-250.
